# A Chromosome-Level Genome Assembly of the Mandarin Fish (*Siniperca chuatsi*)

**DOI:** 10.3389/fgene.2021.671650

**Published:** 2021-06-23

**Authors:** Weidong Ding, Xinhui Zhang, Xiaomeng Zhao, Wu Jing, Zheming Cao, Jia Li, Yu Huang, Xinxin You, Min Wang, Qiong Shi, Xuwen Bing

**Affiliations:** ^1^Key Laboratory of Freshwater Fisheries and Germplasm Resources Utilization, Ministry of Agriculture, Freshwater Fisheries Research Center, Chinese Academy of Fishery Sciences, Wuxi, China; ^2^Shenzhen Key Lab of Marine Genomics, Guangdong Provincial Key Lab of Molecular Breeding in Marine Economic Animals, BGI Academy of Marine Sciences, BGI Marine, Shenzhen, China; ^3^BGI Education Center, University of Chinese Academy of Sciences, Shenzhen, China; ^4^BGI Zhenjiang Institute of Hydrobiology, Zhenjiang, China

**Keywords:** the mandarin fish (*Siniperca chuatsi*), whole-genome sequencing, feeding habit, molecular mechanism, chromosome-level genome assembly

## Abstract

The mandarin fish, *Siniperca chuatsi*, is an economically important perciform species with widespread aquaculture practices in China. Its special feeding habit, acceptance of only live prey fishes, contributes to its delicious meat. However, little is currently known about related genetic mechanisms. Here, we performed whole-genome sequencing and assembled a 758.78 Mb genome assembly of the mandarin fish, with the scaffold and contig N50 values reaching 2.64 Mb and 46.11 Kb, respectively. Approximately 92.8% of the scaffolds were ordered onto 24 chromosomes (Chrs) with the assistance of a previously established genetic linkage map. The chromosome-level genome contained 19,904 protein-coding genes, of which 19,059 (95.75%) genes were functionally annotated. The special feeding behavior of mandarin fish could be attributable to the interaction of a variety of sense organs (such as vision, smell, and endocrine organs). Through comparative genomics analysis, some interesting results were found. For example, olfactory receptor (OR) genes (especially the beta and delta types) underwent a significant expansion, and endocrinology/vision related *npy*, *spexin*, and *opsin* genes presented various functional mutations. These may contribute to the special feeding habit of the mandarin fish by strengthening the olfactory and visual systems. Meanwhile, previously identified sex-related genes and quantitative trait locis (QTLs) were localized on the Chr14 and Chr17, respectively. 155 toxin proteins were predicted from mandarin fish genome. In summary, the high-quality genome assembly of the mandarin fish provides novel insights into the feeding habit of live prey and offers a valuable genetic resource for the quality improvement of this freshwater fish.

## Introduction

The mandarin fish, *Siniperca chuatsi*, belonging to the family Percichthyidae and order Perciformes, has a relatively high market value and widespread aquaculture throughout China (Liang and Cui, 1982; Liu et al., 1998). It has a special feeding habit, accepting only live prey fishes and refusing dead food items in the wild (Chiang, 1959; Liu et al., 1998). The feeding behaviors of the mandarin fish require interactions of a variety of sense organs, such as eyes, mouth, lateral lines, and olfactory organs. Lateral-line may help alert the fish to vibrations that are made by nearby prey or approaching predators (Engelmann et al., 2000). Although the mandarin fish can feed properly on live prey fishes depending mainly on eyes and lateral-line, it can hunt prey fishes without these two organs (Liang et al., 1998). Researchers observed that the mandarin fish could recognize prey fishes using vision (Wu, 1988). A previous study (Liang et al., 1998) reported that the mandarin fish usually stayed more frequently near a perforated opaque cylinder containing live prey fishes rather than those without prey fishes, suggesting the importance of olfaction in searching for prey. However, this conclusion did not justify the food smells from other stimuli (such as hydromechanical stimulus).

Fish toxins have been poorly studied compared to venoms from other animals such as snakes, scorpions, spiders, and cone snails (Utkin, 2015). It is estimated that there are up to 2,900 venomous fishes (Xie et al., 2017) with venom systems convergently evolved 19 times (Harris and Jenner, 2019). Mandarin fish is one of those who can produce toxins in their hard spines to help them defense and prey, and cause pain and swelling at the site of the sting in human as well (Zhang F.-B. et al., 2019). However, apart from several antimicrobial peptides that can be regarded as toxins (Sun et al., 2007), there is no detailed report on venom genes and components of this fish yet.

In Mandarin fish species, females grow faster than males. Whether female mandarin fish have stronger predation ability is still unknown, so gender screening is of great significance to the cultivation of mandarin fish. So far, several gender-related molecular markers or functional genes have been screened, and even all-female mandarin fish have been bred (Guo et al., 2021; Liu et al., 2021). However, due to the lack of available genomic and transcriptome information, the mechanisms of sex differentiation remain poorly understood.

By far, genome data of the mandarin fish have been limited, which restricts genetic information for functional genomics studies. Therefore, in this study, we report a chromosome-level genome assembly of the mandarin fish using a combination of next-generation sequencing and previously reported genetic linkage map. The subsequent comparative genomic analysis provides novel insights into the feeding habit of live prey, toxin, and sex differentiation in the mandarin fish. This genome can not only serve as the genetic basis for in-depth investigations of fish evolution and biological functions but also offers a valuable genetic resource for quality improvement of this economically important fish.

## Materials and Methods

### Sample Collection, Library Construction, and Sequencing

**FIGURE 1:**
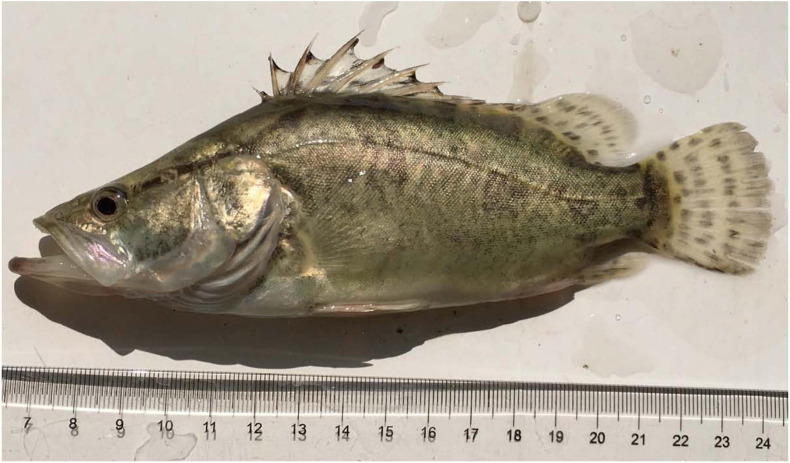
The sampled mandarin fish (*Siniperca chuatsi*).

We collected muscle samples and extracted genomic DNA from a mandarin fish (Figure 1), which was obtained from Freshwater Fisheries Research Center of Chinese Academy of Fishery Sciences, Wuxi City, Jiangsu Province, China. The extracted DNA was used to construct seven libraries, including three short-insert (270, 500, and 800 bp) and four long-insert (2, 5, 10, and 20 kb) libraries. Subsequently, applying the routine whole-genome shotgun sequencing strategy, we sequenced these libraries on a Hiseq2500 platform (Illumina, San Diego, CA, United States). Those raw reads with adapters or low-quality sequences were filtered by a SOAPfilter (v2.2) (Luo et al., 2015).

All experiments were carried out following the guidelines of the Animal Ethics Committee and were approved by the Institutional Review Board on Bioethics and Biosafety of BGI, China (No. FT 18134).

### Estimation of the Genome Size and Generation of a Genome Assembly

We performed a 17-mer distribution analysis to estimate the target genome size using the clean reads from the short-insert libraries (Liu et al., 2013). The calculation of genome size was based on the following formula: G = knum/kdepth. Here, knum is the sequenced k-mer number and kdepth is the k-mer sequencing depth. We set optimized parameters (pregraph-K 41 –d 1; contig –M 1; scaff –b 1.5) for the SOAPdenovo2 software (v2.04) to generate contigs and original scaffolds (Luo et al., 2012). Subsequently, we employed GapCloser (v1.12; with parameter settings of −t 8 −l 150) to fill the gaps of intra-scaffolds (Li et al., 2009) using the clean reads from short-insert libraries (270, 500, and 800 bp). Finally, we used BUSCO (Benchmarking Universal Single-Copy Orthologs; v1.22) to assess genome integrity (Simao et al., 2015).

### Repeat Annotation, Gene Prediction, and Functional Annotation

Repetitive sequences including tandem repeats and transposable elements (TEs) were predicted from the assembled genome. We used Tandem Repeats Finder (v4.07) to search for tandem repeats (Benson, 1999). RepeatMasker (v4.0.6) and RepeatProteinMask (v4.0.6) were employed to detect known TEs based on the Repbase TE library (Tarailo-Graovac and Chen, 2009; Bao et al., 2015). Besides, we used RepeatModeler (v1.0.8) and LTR_FINDER (v1.0.6) with default parameters to generate the *de novo* repeat library (Xu and Wang, 2007; Abrusan et al., 2009), and RepeatMasker was applied to search the repeat regions against the built repeat library.

We utilized three different approaches to annotate structures of predicted genes in our assembled genome, including *de novo* prediction, homology-based prediction, and transcriptome-based prediction. For the *de novo* prediction, we employed AUGUSTUS (v3.2.1) and GENSCAN (v1.0) to identify protein-coding genes within the mandarin fish genome, using the repeat-masked genome as the template (Burge and Karlin, 1997; Stanke et al., 2006). For the homology-based prediction, we aligned the homologous proteins of five other fish species, including zebrafish (*Danio rerio*), three-spined stickleback (*Gasterosteus aculeatus*), Nile tilapia (*Oreochromis niloticus*), medaka (*Oryzias latipes*), and fugu (*Takifugu rubripes*) (downloaded from Ensembl 83 release), to the repeat-masked genome using tblastn (v2.2.26) with an E-value ≤ 1e-5 (Mount, 2007; Cunningham et al., 2015). Subsequently, Solar (v0.9.6) and GeneWise (v2.4.1) (Birney and Durbin, 2000) were executed to define the potential gene structures for all alignments. The RNA-seq data from muscle tissues were aligned to the assembled genome also using tophat (v2.0.13) (Trapnell et al., 2009) and gene structures were predicted using cufflinks (v2.1.1) (Trapnell et al., 2012). Finally, we combined the above-mentioned three datasets to obtain a consistent and comprehensive gene set by GLEAN (v1.0) (Elsik et al., 2007).

These predicted coding proteins of the mandarin fish were aligned against public gene ontology (GO), Kyoto Encyclopedia of Genes and Genomes (KEGG), Swiss-Prot and TrEMBL databases for annotation of functions and pathways by using BLASTP (Kanehisa and Goto, 2000; Boeckmann et al., 2003; Harris et al., 2004). Subsequently, we applied InterProScan (v5.16−55.0) to identify functional motifs and domains through Pfam, PRINTS, ProDom, and SMART databases (Attwood, 2002; Letunic et al., 2004; Bru et al., 2005; Hunter et al., 2009; Finn et al., 2014).

### Genome Evolution Analysis

Protein sequences of five ray-fin fishes, including spotted gar (*Lepisosteus osseus*), zebrafish, three-spined stickleback, medaka, and fugu were downloaded from the Ensembl (release-83) (Cunningham et al., 2015). Protein sequences of Asian arowana (*Scleropages formosus*, assembly fSclFor1.1) and giant-fin mudskipper (*Periophthalmus magnuspinnatus*, assembly fPerMag1.pri) were obtained from our recent works (You et al., 2014; Bian et al., 2016). Subsequently, OrthoMCL (v1.4) was executed to cluster the predicted mandarin fish genes into families (Li et al., 2003). We then identified and selected one-to-one orthologs from the above-mentioned eight teleost species, and finally used MUSCLE (v3.8.31) to perform multiple sequence alignment and PhyML (v3.0) to construct a phylogenetic tree (Edgar, 2004; Guindon et al., 2009, 2010).

### Pseudo-Chromosome Construction

Single nucleotide polymorphisms (SNPs)-containing reads in the genetic linkage map of *S. chuatsi* (Sun et al., 2017) were mapped to our assembled mandarin fish genome, and the best hit reads were selected. Linkage groups (LGs) were assigned using the JoinMap4.1 software (Van Ooijen, 2006). Subsequently, a genetic linkage map of the mandarin fish was reconstructed, and SNPs in the genetic linkage map were used for assembling chromosomes. Based on genetic distances between these SNP markers, we determined the position and orientation of each scaffold and then anchored these scaffolds to construct pseudo-chromosomes.

To perform the genome synteny analysis, we downloaded genome sequences of European sea bass (*Dicentrarchus labrax*) from NCBI (Tine et al., 2014) as a reference. Genome-wide alignments were performed using lastz (Kurtz et al., 2004), and the best homology segments were selected using perl scripts. The final genomic synteny was visualized using the Circos software (Krzywinski et al., 2009).

### Localization of Sex-Related Genes and Quantitative Trait Locis on Chromosomes

To identify candidate genes for underlying sex dimorphisms, we downloaded 81 putative sex-related genes from NCBI (Eshel et al., 2012, 2014; Zeng et al., 2016). The distribution of sex-related genes on chromosomes were determined by homologous sequence alignment.

### Identification of *leptin*, Neuropeptide (*npy*), and *spexin* Genes From Teleost Fish Genomes

Protein sequences of three food intake genes, including leptin (NP_001122048.1), npy (AAI62071.1) and spexin (XP_010740053.1) protein sequences were downloaded from NCBI. We performed tblastn (v2.26) (Mount, 2007) with default parameters searching against the mandarin fish, large yellow croaker (*Pseudosciaena crocea*; assembly L_crocea_2.0), grass carp (*Ctenopharyngodon idella*; assembly CI01), yellowtail (*Seriola dumerili*; assembly Sdu_1.0), kingfish (*Seriola lalandi*; assembly Sedor1), orange-spotted grouper (*Epinephelus coioides*), sea bass (*Lates calcarifer*; assembly ASM164080v1), and juvenile ovate pompano (*Trachinotus ovatus*; Zhang D.-C. et al., 2019) genome sequences. Subsequently, Genewise (v2.2.0) was applied to extract the best alignment results (Birney et al., 2004).

### Identification of Olfactory Receptor and Taste Receptor Genes From Genome Sequences

We used zebrafish and pufferfish olfactory receptor (OR) protein sequences (Supplementary Table 1) as the queries to extract the OR genes in the Mandarin fish, zebrafish, fugu, stickleback, medaka, giant-fin mudskipper, Asian arowana and spotted gar (following the method mentioned in the previous section).

Protein sequences of five different types of taste receptor (TR) genes, including sour TR genes (*D. rerio*: ENSDARP 00000119061), sweet TR genes (*D. rerio*: NP_001077325.1, NP_001034920.1, and *T. rubripes*: NP_001091094.1), umami TR genes (*D. rerio*: NP_001034614.2, NP_001034717.1 and *T. rubripes*: NP_001098687.1, NP_001072097.1), bitter TR genes (*D. rerio*: ENSDARG00000079880), salty TR genes (*Homo sapiens*: NP_001153048.1, NP_000327.2, and NP_001030.2), were downloaded from the NCBI or Ensembl database. We performed tblastn (Blast v2.26; Pevsner, 2005) with default parameters to search against these genome sequences. Subsequently, Genewise v2.2.0 (Birney et al., 2004) was employed to extract the best alignment results.

Proteins sequences were aligned by the MAFFT (v7.237) program (Katoh et al., 2002) with the einsi module. Phylogenetic trees were constructed using the PhyML (v3.0) program with bootstrap set to 1,000 (Guindon et al., 2010).

### Identification of *opsin* Genes From Ray-Finned Fish Genomes

In this study, we chose eight teleost genomes to extract opsin protein sequences, including the mandarin fish, Asian arowana, mudskipper, spotted gar, medaka, stickleback, fugu, and zebrafish. Protein sequences of opsin genes (LWS-1: ENSDARP00000065940, LWS-2: ENSDARP00000149112, SWS-1: ENSDARP00000067159, SWS-2: ENSDARP00000144766, RH1: ENSDARP00000011562, RH2-1: ENSDARP00000001158, RH2-2: ENSDARP00000011837, RH2-3: ENSDARP00000001943, and RH2-4: ENSDARP000 00000979) from zebrafish were downloaded from the Ensembl database as the queries. We performed tblastn (v2.2.28) (Mount, 2007) to align these sequences. Finally, Exonerate (v2.2.0) (Slater and Birney, 2005) was employed to predict the perfect alignment results. Multiple sequence alignment of these predicted *opsin* genes was performed with the Muscle module in MEGA (v 7.0) (Kumar et al., 2016). They were then translated into protein sequences for phylogenetic analyses. Phylogenetic trees were constructed using the PhyML (v3.0) program with bootstrap set to 1,000 (Guindon et al., 2010).

### Venom Proteins Prediction

Protein sequences of animal venoms and toxins (Supplementary Table 2) were downloaded from UniProtKB/Swiss-Prot (UniProt Consortium, 2018) via the Animal Toxin Annotation Project (Jungo et al., 2012). These protein sequences were then filtered and only reviewed references (7,093 in total) were maintained as the trust-worthy input queries for searching. Firstly, we blasted the reviewed toxins against the coding sequences (CDS) predicted from the mandarin fish genome assembly using blastp (Camacho et al., 2009) with an e-value of 1e-10. Subsequently, the mapped sequences of mostly partial or fragmented genes with aligning ratios less than 75% were discarded, and the remaining 195 hits were further filtered manually according to the constrained lengths of the venom sequences within the same family, conserved patterns (e.g., disulfide bonds) and other post-translational modifications (PTMs).

## Results

### Genome Sequencing and Assembly

Seven libraries including three short-insert (270, 500, and 800 bp) and four long-insert (2, 5, 10, and 20 kb) were constructed to generate a total of 327 Gb raw reads (Supplementary Table 3). Subsequently, these raw data were filtered, and 233 Gb clean data were obtained for subsequent genome assembly.

We calculated the genome size using the following formula: G = knum/kdepth. Here, the knum (i.e., k-mer number) was 43,888,350,480 and the kdepth (k-mer depth) was 59. Therefore, the estimated genome size of the mandarin fish is about 743.87 Mb (Supplementary Table 4 and Supplementary Figure 1).

We generated contigs and original scaffolds by paired-end reads to assemble the mandarin fish genome. After filling the gaps of intra-scaffolds, we obtained a 758.78-Mb genome assembly for the mandarin fish, with contig and scaffold N50 values of 46.11 Kb and 2.64 Mb, respectively (Table 1).

**TABLE 1:** Summary of the mandarin genome assembly and annotation.

Genome assembly	Contig N50 size (kb)	46.11
	Scaffold N50 size (Mb)	2.64
	Assembled genome size (Mb)	758.78
	Genome coverage (X)	430.83
	The longest scaffold (bp)	16,398,010
Genome characteristics	GC content	39.2%
	Gene number	19,904
	BUSCOs (complete in total)	86.1%

Using BUSCO analysis to determine the completeness of our assembly, it is found that the assembly contained 86.1% complete, 3.0% duplicated, 9.3% fragmented, and 3.7% missed BUSCOs. Besides, 74.6% of the clean reads of RNAseq could be mapped to the genome assembly. These results suggested that our genome assembly was relatively complete.

### Chromosome-Level Genome Assembly

**FIGURE 2:**
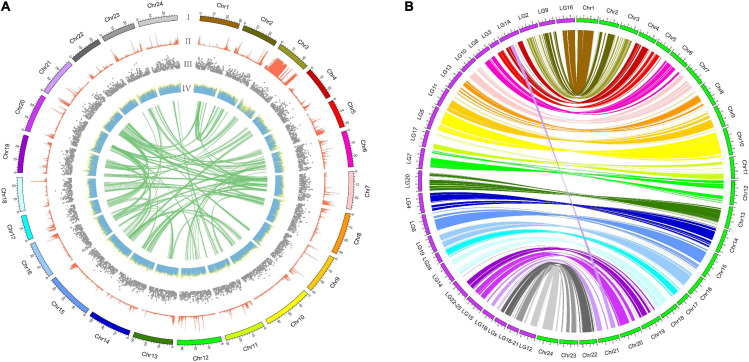
Chromosome-level genome assembly of the mandarin fish. **(A)** A Circos atlas presents details of the pseudo-chromosome information from outside to inside: (I) the length of each pseudo-chromosome, (II) density of SNP distribution in each 100-kb genomic interval, (III) density of gene distribution in each 100-kb genomic interval, (IV) GC content of 100-kb genomic intervals, and (V) schematic presentation of major inter-chromosomal relationships in the mandarin fish genome. **(B)** A synteny comparison between mandarin fish and seabass genomes (chr: mandarin and LG: seabass) revealed high accuracy of our assembled genome for the mandarin fish.

Based on the previously reported genetic linkage map of the mandarin fish (Sun et al., 2017), we anchored a total of 518 scaffolds into 24 chromosomes (Chr). A total of 697.06 Mb was assembled, corresponding to 92.8% of the assembled genome and 18,752 genes (from a total of 19,904 genes). The largest chromosome was Chr10 with 37.87 Mb in length containing 56 scaffolds, and the smallest was Chr17 with 19.19 Mb containing 22 scaffolds. The average chromosome length was 29 Mb with 21 scaffolds (Table 2 and Figure 2A).

**TABLE 2:** Summary of the assembled chromosomes of the mandarin fish.

**Chr**	**Length (Mb)**	**Mapped scaffolds**	**Mapped genes**
Chr1	30.26	14	930
Chr2	27.62	24	750
Chr3	26.07	42	505
Chr4	25.64	23	723
Chr5	22.01	39	480
Chr6	28.28	23	984
Chr7	29.34	35	906
Chr8	32.52	20	834
Chr9	30.54	15	710
Chr10	37.87	15	817
Chr11	30.42	22	843
Chr12	33.69	35	977
Chr13	30.45	16	922
Chr14	32.91	21	892
Chr15	36.19	17	1,030
Chr16	28.49	10	776
Chr17	19.19	22	405
Chr18	26.51	19	706
Chr19	29.45	21	892
Chr20	29.82	19	732
Chr21	29.59	16	960
Chr22	24.33	11	563
Chr23	25.99	23	554
Chr24	29.85	16	861
Total	697.06	518	18,752

There were 39,689 synteny blocks (>2 kb) between the assembled genomes of the mandarin fish and the reported European sea bass (Tine et al., 2014). We observed that almost all chromosomes showed the 1:1 synteny relationship, with an exception of Chr21 in the mandarin fish that aligned to two seabass chromosomes (Figure 2B). The results of collinearity between mandarin fish and European sea bass indicate that our chromosome assembly results are reliable.

### Genome Annotation

Repeat sequences were identified based on homology search against the Repbase database and *de novo* prediction. We predicted that the mandarin fish genome contained 26.3% of repetitive elements. Compared with other perciforme fish, the mandarin fish was lower than red-spotted grouper (43.02%), giant grouper (45.1%), but much higher than large yellow croaker (18.1%) and golden pompano (20.25%) in the repeat sequence percentage. The most abundant TEs were long interspersed elements (13.96% of the genome), followed by DNA transposons (9.66%) and long terminal repeats (LTRs, 5.04%) (Supplementary Table 5).

Based on the genome with repeated elements masked, we integrated homology searching, *de novo*, and transcript methods to predict that the mandarin fish genome had 19,904 protein-coding genes (Supplementary Table 6), of which 19,059 (95.75%) genes were functionally annotated by at least one of the InterPro, GO, KEGG, Swiss-Prot, and TrEMBL protein databases (Supplementary Table 7). To estimate the completeness of our annotated genes, we determined that the annotated genes contained 88.9% complete, 3.2% duplicated, 6.5% fragmented, and 4.6% missed BUSCOs.

### Phylogenetic Analysis

**FIGURE 3:**
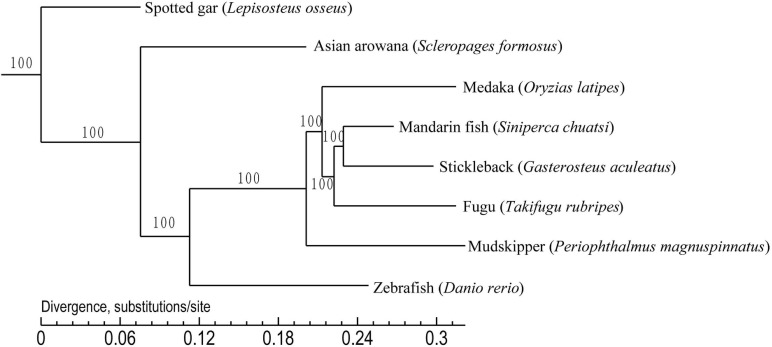
Phylogenetic tree of eight teleost fishes. It was constructed using the shared 3,510 single copy orthologous genes. Spotted gar was used as the outgroup.

To establish the phylogenetic position of the mandarin fish, we compared the genomes of the mandarin fish and seven other teleost fishes. We found that 16,922 orthologous gene families were shared among the eight teleost fishes, and identified 3,510 single-copy orthologs genes that were used to construct a phylogenetic tree (Figure 3). It appears that stickleback was most closely related to the mandarin fish. We selected the specific gene family in mandarin fish and they were functionally annotated by KEGG protein database (Supplementary Table 8). Finally, there were 74 specific families in mandarin fish, containing 194 genes. According to KEGG annotation, there were 45 genes associated to 205 pathways.

### Localization of Sex-Related Genes and QTLs on Chromosomes

**FIGURE 4:**
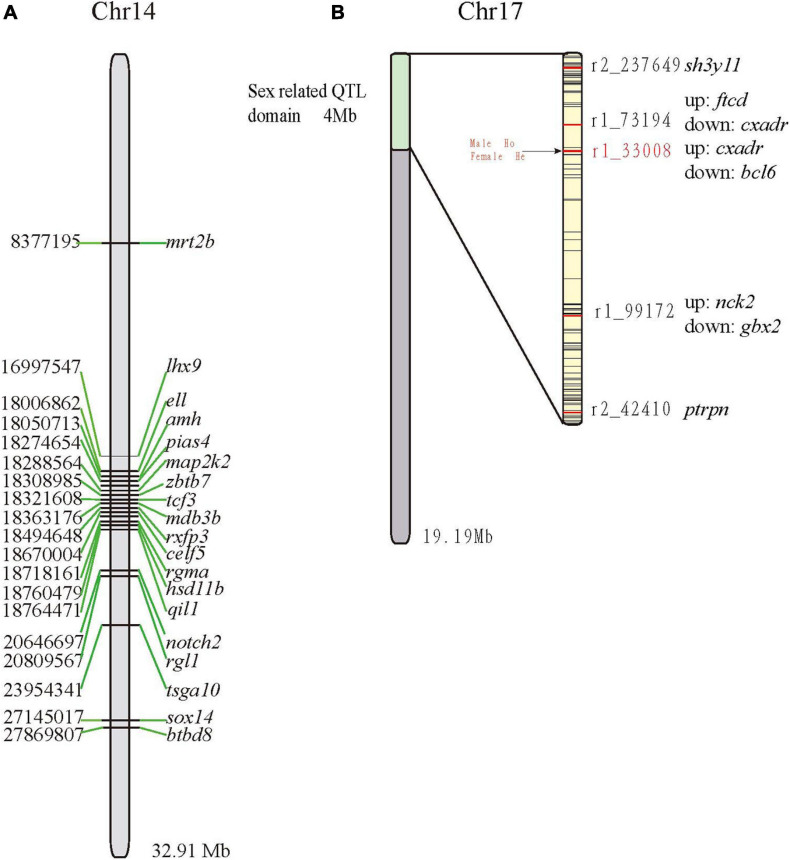
Localization of sex-related genes and QTLs on the Chr14 **(A)** and Chr17 **(B)**, respectively. Abbreviations of genes: *amh*, anti-mullerian hormone; *btbd8*, BTB (POZ) domain containing 8; *celf5*, CUGBP Elav-like family member 5; *dmrt2b*, doublesex and mab-3 related transcription factor 2b; *ell*, RNA polymerase II elongation factor ELL; *hsd11b*, hydroxysteroid 11-beta-dehydrogenase 1-like protein; *lhx9*, LIM homeobox 9; *map2k2*, dual specificity mitogen-activated protein kinase kinase 2; *notch2*, notch homolog 2; *pias4*, Protein inhibitor of activated STAT 4; *qil1*, QIL1; *rgl1*, ral guanine nucleotide dissociation stimulator-like 1; *rxfp3*, relaxin family peptide receptor 3; *tcf3*, E2A-1 transcription factor; sox14, SRY-box transcription factor; *zbtb7*, zinc finger and BTB domain containing 7.

Of the 81 sex-related genes, 19 genes were located on Chr14 (13 clustered in Figure 4A). In a previous study (Sun et al., 2017), five QTLs for sex determination (SD) were detected on LG23 (Sun et al., 2017) and thereby localized on Chr17 of the mandarin fish genome (clustered between 0 and 4 Mb; Figure 4B). Both r2_42410 and r2_237649 were located within the receptor-type tyrosine-protein phosphatase-like N (*ptrpn*) and SH3 domain-containing YSC84-like protein 1 (*sh3yl1*), respectively. The other three QTLs were located in the intergenic regions (Figure 4B). Genotypes of all the male and female fishes on r1_33008 were homozygous and heterozygous respectively, which was reported previously (Sun et al., 2017). Subsequently, we validated the marker r1_33008 (Sun et al., 2017) in another group (Figure 4B) and found that there was no difference between male and female, which may be unique in the genetic linkage map population.

### Analysis of Genes for Food Intake

In fishes, feeding behaviors are usually regulated by specific regions in the brain, the so-called feeding centers, which are under the influence of hormones produced by the brain and the periphery (Volkoff, 2016). The mandarin fish has a peculiar feeding habit of only accepting live prey fishes and refusing artificial diets or dead prey fishes. It is almost unknown about any genes for regulation of this unique food preference (Liu et al., 1998; Li et al., 2013). In our present study, several candidate genes for food intake were analyzed.

**FIGURE 5:**
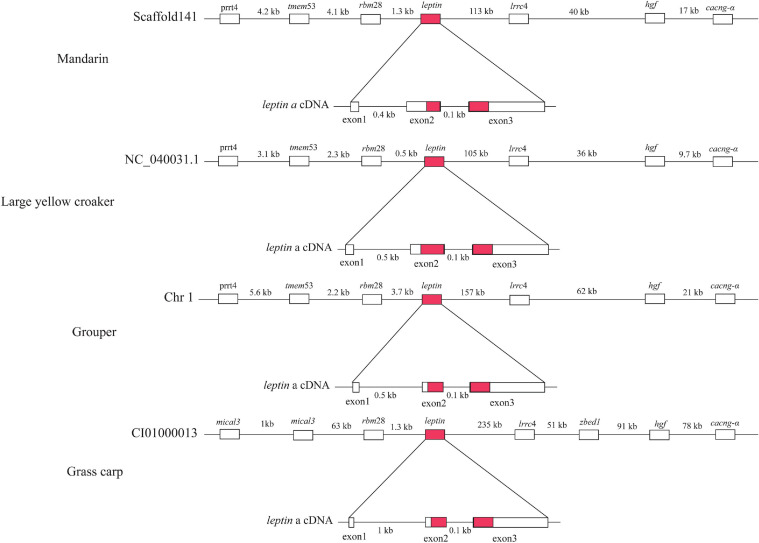
Comparison of *leptin* gene structures among mandarin fish, large yellow croaker, grouper and grass carp. Red marked areas represent open reading frames.

*leptin* is an important hormone involved in the regulation of food intake and energy balance (Kurokawa et al., 2005). Our synteny analysis of four representative fishes (mandarin, large yellow croaker, grouper and grass carp) (Figure 5) indicated that the upstream and downstream of the *leptin* genes are proline-rich transmembrane protein 4 (*prrt4*), transmembrane protein 53 (*tmem53*), RNA-binding protein 28 (*rbm28*) and Leucine-rich repeat-containing protein 4 (*lrrc4*), hepatocyte growth factor (*hgf*), voltage-dependent calcium channel subunit alpha (*cacng-*α) genes respectively, which is consistent with a previous report (Kurokawa et al., 2005). Compared with the other three species, the upstream genes {[F-actin]-monooxygenase MICAL3 (*mical3*) and zinc finger BED domain-containing protein 1 (*zbed1*)} of grass carp were not conserved (Figure 5). In a previous study (He et al., 2013), a typical *leptin* gene was reported to be composed of three exons and two introns, but the mandarin fish *leptin* gene consisted of two exons and one intron. However, our genomic results confirmed that the *leptin* structure of the mandarin fish is in fact consistent with the other three representative fishes (grouper, large yellow croaker, and grass carp) (Supplementary Figure 2). We thereby propose that the errors in the previous study are due to a shortage of whole genome sequence of the mandarin fish at that time.

Neuropeptide Y (*npy*), belonging to the *npy* family, is abundant in the central nervous system (Volkoff, 2006; Holzer et al., 2012). *npy* has been implicated in several centrally mediated physiological functions, such as regulation of body temperature, sexual behavior, energy homeostasis, anxiety, mood, and neuroendocrine secretions (Holzer et al., 2012). Moreover, *npy* is one of the most abundant neuropeptides within the brain and has a major regulatory role in food intake (Yokobori et al., 2012; Zhou Y. et al., 2013). The *npy* gene of the mandarin fish on Chr 2 was comprised of three exons and two introns, which is consistent with other fishes. Mandarin fish compared to six perciforme fishes, NH2-terminal signal peptide (red box, Supplementary Figure 3) is variable, mature peptide (Black underline, Supplementary Figure 3) is highly conserved. However, its COOH-terminal domain had two significant variant sites (red asterisks in Supplementary Figure 3).

*Spexin* was identified in mammalian adipose tissue. It plays a significant role in the regulation of energy metabolism and food intake (Walewski et al., 2014; Zheng et al., 2017). Its expression is up-regulated in food deprivation and down-regulated in obese rats and humans, suggesting suppression of the orexin in the hypothalamus (Li et al., 2016). In the present study, we performed sequence analysis and found that the *spexin* gene is composed of six exons and five introns, and the amino acids of the mature peptide (spexin-14; Supplementary Figure 4a) in the mandarin fish are identical to that of the grouper (Li et al., 2016). Sequence alignment of mandarin fish *spexin* with the other six perciforme fishes reveals that the NH_2_-terminal signal peptide is highly variable, and its COOH-terminal domain had two significant variant sites (red asterisks). In contrast, the region covering the *spexin* mature peptide (black box) together with the dibasic processing sites flanking the two ends (RR and GRR, black triangle) is highly conserved (Supplementary Figure 4b).

### Olfactory Receptor Genes in Teleost Fishes

**FIGURE 6:**
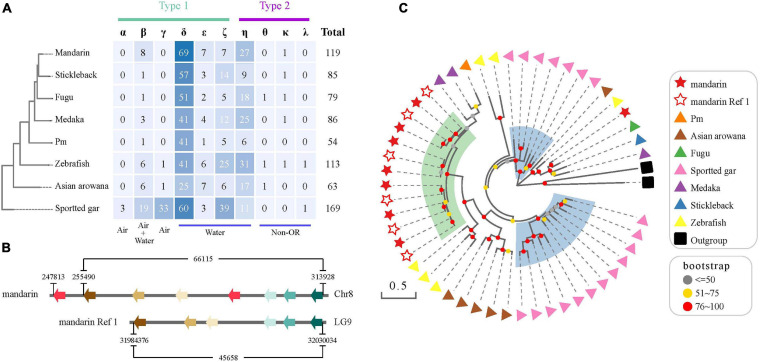
The OR gene family in the mandarin fish genome. **(A)** OR genes in the mandarin fish in comparison with seven other teleost fishes. “Air” and “water” represent the detection of airborne and water-soluble odorants, respectively. **(B)** A comparative genomic analysis of Group β OR functional genes in our genome assembly and the assembly of Ref 1. **(C)** A phylogenetic tree of Group β functional genes in the mandarin fish and seven other teleost fishes. Stars indicate the Group β OR functional genes of the mandarin fish [from our present work and Niimura and Nei (2005)]. Triangles with different colors represent various resources of the other seven teleost fishes. Green transparent rainbow indicates an expansion of Group β OR functional genes in the mandarin fish. Blue transparent rectangles indicate the expansion of Groupe β OR functional genes in spotted gar.

We identified 133 OR genes in the mandarin fish genome (Figure 6A), including 119 functional genes and 13 pseudogenes. The numbers are different from those reported in zebrafish (102 functional genes and 35 pseudogenes) and pufferfish (44 functional genes and 54 pseudogenes) genomes (Niimura and Nei, 2005). We examined zebrafish (Ensembl version: GRCz11) and fugu genomes (Ensembl version: FUGU5) and identified 109 functional genes and 6 pseudogenes in the zebrafish and 72 functional genes and 12 pseudogenes in the fugu, respectively.

We found that the mandarin fish had more OR functional genes than other examined teleosts, except for the spotted gar that was diverged from teleosts before the teleost-specific genome duplication (TGD). Spotted gar has 36 functional genes ascribing to Groups α and γ, which were mostly absent in teleosts. The mandarin fish had the largest numbers of Group β (*n* = 8) and group δ (*n* = 69) functional genes among other teleosts. In a previous study (Lv et al., 2019), researchers indicated an expansion of Group β OR genes in the mandarin fish, which was confirmed in our present work (Figure 6C). We extracted more Group β OR genes (eight in Chr8) than a previous report (six in Reference (Niimura and Nei, 2005); see Figures 6B,C).

### Taste Receptor Genes in Teleost Fishes

**FIGURE 7:**
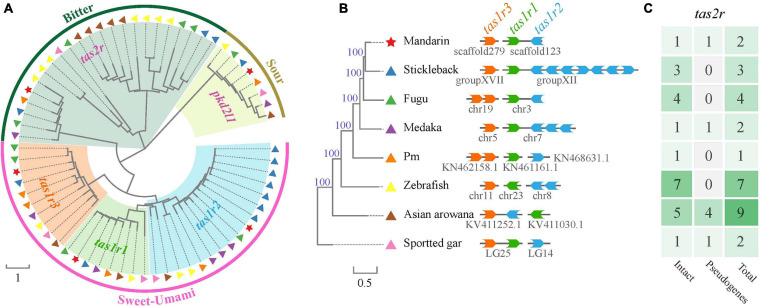
A phylogenetic tree of taste-related genes in the mandarin fish and seven other teleost fishes. **(A)** Comparisons of taste-related genes in the mandarin fish and seven other teleost fishes. Red stars indicate the gens of the mandarin fish. Other symbols indicate the sources of other species. **(B)** Genomic locations of *tas1r1*, *tas1r2*, and *tas1r3* genes in our genome assembly. **(C)** Summary of the copy number of *tas2r* gene in different species [corresponding to those fishes in panel **(B)**].

Taste receptor type 1family (*tas1r*), belonging to the G protein-coupled receptor (*gpcr*), plays a central role in the reception of sweet and umami taste in many vertebrates. A *tas1r2* + 3 heterodimer was identified as the sweet TR (Nelson et al., 2001; Li et al., 2002). Tas1r3 may serve as a receptor for high sucrose concentrations (Zhao et al., 2003). A *tas1r1* + 3 heterodimer and multiple combinations of *tas1r2* with *tas1r3* were identified as a tuned L-amino acid TR in fish (Oike et al., 2007). We extracted three *tas1r* genes in the mandarin fish, Asian arowana and spotted gar (Table 3 and Figure 7). It seems that the gene numbers responding to sweet and umami tastes in the mandarin fish is more primitive since they are much closer to ancient fishes (such as arowana and gars).

**TABLE 3:** Numbers of taste receptor genes in these species.

**Group**	**Mandarin**	**Zebrafish^*I*^**	**Fugu^*II*^**	**Stickleback**	**Medaka**	**PM**	**Arowana**	**Spotted gar**
Sour	1	1(1)	1	1	1	1	1(1)	1(2)
Sweet-Umami	3	4	4	9	5	4	3	3
Bitter	1(1)	7	4	3	1(1)	1	5(4)	1(1)
Salty	0	0	0	0	0	0	0	0
Total	5(1)	12(1)	9	13	7(1)	6	9(5)	5(3)

*^*I*^The version of zebrafish genome is GRCz11.^*II*^The version of fugu genome is FUGU5.*

Bitter taste preference was likely recognized as a mechanism for avoiding toxic foods. Bitter foods evoke innate aversive behaviors in many animals. Taste receptor type 2 (*tas2r*) family was identified as bitter TRs in mammals. Most vertebrate species have several *tas2r* genes, and their copy numbers varied among various species. In our present study, we identified only one intact *tas2r* gene in the mandarin fish, giant-fin mudskipper, and spotted gar (Figure 7C), suggesting that these three species possibly have a low ability to distinguish the bitter foods. However, compared with the other seven teleost fishes, the mandarin fish showed no difference in number of genes for responding to salty and sour tastes (Table 3).

### Comparison of Mandarin Fish *opsin* Genes With Other Teleost Fish

**FIGURE 8:**
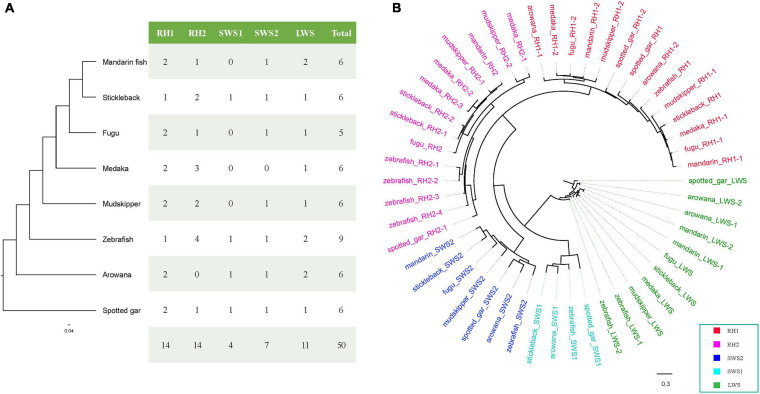
o*psin* genes in the mandarin fish genome. **(A)** *opsin* genes in the mandarin fish and seven other teleost fishes. **(B)** A phylogenetic tree of *opsin* genes in the mandarin fish and seven other teleost fishes. LWS, long wavelength-sensitive; RH1, rhodopsin; RH2, green-sensitive; SWS1, short wavelength-sensitive 1; SWS2, short wavelength-sensitive 2.

A total of 50 *opsin* nucleotide sequences, including 14 *RH1* (rhodopsin), 14 *RH2* (green-sensitive), 4 *SWS1* (short wavelength-sensitive 1), 7 *SWS2* (short wavelength-sensitive 2), and 11 *LWS* (long wavelength-sensitive), were successfully derived from eight teleost fishes (Figure 8A). To understand the evolutionary relationships among these opsins in teleosts, we constructed a phylogenetic tree (Figure 8B) using spotted gar LWS as the outgroup. Obviously, all opsin could be divided into five main clades (RH1, RH2, SWS1, SWS2, and LWS) in the eight examined teleost species.

In this study, we identified six *opsin* genes in the mandarin fish genome (Figure 8A), including two *RH1*, one *RH2*, one *SWS2*, and one *LWS*. A previous study (Neafsey and Hartl, 2005) reported loss of *SWS1* genes in fugu and mudskipper (You et al., 2014) genome. We tried to extract *SWS1* sequences in the examined teleosts, but could not find *SWS1* in the mandarin fish, medaka, fugu, and giant-fin mudskipper either. This loss of *SWS1* could be an adaptation to minimize retinal damage from ultraviolet. We identified two *LWS* genes in the mandarin fish, zebrafish and arowana, while only one *LWS* in other fishes. In zebrafish, two *LWS* genes locating in tandem encode different protein sequences (Chinen et al., 2003). However, in the mandarin fish and arowana, two *LWS* genes were also in tandem but encoded the identical protein sequences. According to Figure 8B and Supplementary Figure 4, m and arin LWS-1 and LWS-2 were completely same in amino acid sequences. And arowana LWS-1 and LWS-2 had same sequences but they were different in zebrafish.

The primary amino acid sequence is very important for *opsin* molecular properties. In this study, we compared their sequences and identify the differences among the eight examined fishes. We identified some amino acid changes in the mandarin fish that are probably critical for wavelength absorption. Here, we observed five specific sites in the mandarin fish, with significant differences from the other seven fish species (see more details in Supplementary Figure 5). We identified the transmembrane domains of *LWS* with TMHMM Server (Version 2.0)^1^ (Supplementary Figure 5). In the mandarin fish, two specific sites are in the transmembrane domains, L98 and T219, which are potentially important for light absorption. *LWS* is often used for red vision, and shallow water receives more red light. The freshwater fishes have more *LWS* genes than those in seawater (Lin et al., 2017). The more *LWS* genes and several specific sites in transmembrane domains help fishes be more sensitive to light and live prey.

### Toxin Genes Were Identified in the Mandarin Fish Genome

Fish toxins have been poorly studied compared to venoms from other animals such as snakes, scorpions, spiders, and cone snails (Utkin, 2015). It is estimated that there are up to 2,900 venomous fishes (Xie et al., 2017) with venom systems convergently evolved 19 times (Harris and Jenner, 2019). Mandarin fish is one of those who can produce toxins in their hard spines to help them defense and prey, and cause pain and swelling at the site of the sting in human as well (Zhang F.-B. et al., 2019). However, apart from several antimicrobial peptides that can be regarded as toxins (Sun et al., 2007), there is no detailed report on venom genes and components of this fish yet.

In this study, a total of 155 toxin proteins were predicted from the mandarin fish genome assembly. They ranged from 87 to 1,895 amino acids (aa), with more than half of them less than 300 aa (Supplementary Figure 6). Unlike a vast number (125) of short-length “fragmented” venoms (less than 100 aa) in the Chinese yellow catfish genome (Zhang et al., 2018), there were only two short-length venoms in the mandarin genome, with a length of 87 and 98 aa, respectively. Consistent with the common findings that most toxins are short peptides, the majority (96; 62%) of our predicted toxin proteins had an entire length between 100 and 300 aa.

Among the 155 putative venom proteins, 144 were classified into 37 families, with 11 unclassified toxins (Supplementary Figure 7). The top four biggest groups in these toxins included peptidase S1, venom metalloproteinase (M12B), Type-B carboxylesterase, and calmodulin, consisting of 27, 13, 10, and 9 toxin genes respectively. Interestingly, several fish-specific toxins were identified, including SC_GLEAN_10016806 and SC_GLEAN_10016808 that belonged to the stonustoxin (SNTX-a), SC_GLEAN_10016805, and SC_GLEAN_10016807 annotated as SNTX-β. SNTX is a soluble heterodimeric assembly of α and β subunits that share a sequence identity of ∼50% (Ellisdon et al., 2015). It was firstly isolated from the stonefish (Poh et al., 1991) and has been proved to induce platelet aggregation and hemolytic activity (Khoo et al., 1992) and also function as a neurotoxin (Low et al., 1994). The existence of two copies of both SNTX-α and SNTX-β suggesting the probability of the forming of active and functional toxins in the mandarin fish. SNTXs, along with all other toxins identified in this assembled genome, showed the great potential of discovering new drugs.

## Discussion

Our high-quality genome assembly of the mandarin fish could provide opportunities to understand SD, special food intake, or other biological processes at the genome level in this economically important fish. The final assembled genome was 758.78 Mb, and approximately 92.8% of the scaffolds were ordered onto 24 chromosomes. The mandarin fish has been widely cultivated in China, with a special feeding habit of accepting only live prey fishes for its delicious meat. However, little is currently known about related genetic mechanisms. Uncovering the molecular mechanisms for regulation of feeding behaviors may not only lead to specific adjustments in fish culture conditions and feeding strategies but also gradually instruct us to develop new technologies to improve feeding, food conversion efficiency and the growth of aquaculture fishes (Volkoff et al., 2010; Zhou Y. et al., 2013).

In fact, feeding is a complex of behaviors, including at least food intake itself and foraging or appetite behavior. Eating is ultimately regulated by the central feeding center in the brain (Keen-Rhinehart et al., 2013; Woods et al., 2014). It also processes information from endocrine signals from the brain and the surrounding environments. These endocrine signals include various hormones. For example, *npy* and *spexin* are two important hormones involved in the regulation of food intake and energy balance (Zheng et al., 2017; Zhou Y. et al., 2013). The latest research suggests that smell might regulate appetite through *npy* in yellowtail (Senzui et al., 2020). *npy* as a neuromodulator in the olfactory epithelium and intensified the activity of OR neurons and olfaction (Negroni et al., 2012; Senzui et al., 2020). In our present study, we observed that the amino acid sequence of *npy* and *spexin* showed a high level of conservation, when compared with the other six examined Perciformes fishes. It seems that *npy* and *spexin* are conserved neuropeptides in fish evolution with important physiological functions. However, the *npy* and *spexin* genes of the mandarin fish had significant variations at the C-termini of the protein sequences (Supplementary Figures 2, 3), which may be related to the special diet of the mandarin fish.

Olfaction is also crucial for animals to find foods and to judge whether potential foods are edible or not (Chandrashekar et al., 2000; Nei et al., 2008). It is controlled by a large family of OR genes. Fishes also have this gene family, but the number of genes is much less than mammals (Niimura and Nei, 2006). Previous studies have demonstrated that the beta type OR genes are presented in both aquatic and terrestrial vertebrates, indicating that these receptors detect both water-soluble and airborne odorants (Niimura, 2009); however, delta type OR genes are only in aquatic organisms (You et al., 2014). In the present study, we determined that the mandarin fish had the largest numbers of Group β (*n* = 8) and group δ (*n* = 69) functional genes than the other teleost fishes (Figure 6A), which might contribute to its particular carnivorous diet.

Vision is very important for animals because it plays important roles in foraging, mating, information transmission, and escaping from predators (Yokoyama, 2000). Based on their amino acid compositions, *opsin* genes are classified into five common clusters: *RH1* (rhodopsin), *RH2* (rhodopsin-like or the green light-sensitive pigments), *SWS1* (short wavelength−, or the UV or violet light-sensitive pigments), *SWS2* (*SWS1*-like or the blue light-sensitive pigments); *LWS/MWS* (long wavelength- or middle wavelength-sensitive, or the red- and green-sensitive pigments) (Yokoyama, 2000; Shichida and Matsuyama, 2009). *opsin* diversity is usually generated by gene duplication and/or accumulation of mutations. *MWS/LWS* opsins have peak values of light absorption (Terai et al., 2002). The light sensitivity of a visual pigment is determined not only by the chromophore itself, but also by its interaction with the amino acid residues lining the pocket of the opsin (Yokoyama, 1995). In this study, compared with other closely related species, the mandarin fish was identified with more *LWS* genes. *LWS1/2* had five specific sites in the mandarin fish with remarkable differences from the other seven fish species (Supplementary Figure 5). Certain mutations of the transmembrane domains, L98 and T219 in the *LWS* genes might be expected to contribute to the special feeding habit of live prey.

Many fish species exhibit sexual dimorphisms, such as Japanese flounder (*Paralichthys olivaceus*) (Shao et al., 2015), half-smooth tongue sole (*Cynoglossus semilaevis*) (Song et al., 2012), displaying significant differences in growth rates or sizes between male and female individuals. Females of mandarin fish present higher growth rates (by 10–20% in body weight) than males (Sun et al., 2017). Therefore, screening of sex-related genes or markers is important for the development of the mandarin industry, which will be helpful for the elucidation of the SD mechanisms in the mandarin fish. Nineteen sex-related genes, localized on the Chr14, were previously reported to be involved in spermatogenesis, SD, and testicular determination. Some studies support that SD is controlled by many major genetic factors that may interact with minor genetic factors, thereby implying that SD should be analyzed as a quantitative trait (Eshel et al., 2012). Five sex-related QTLs in the mandarin fish were previously detected on the Chr17. Therefore, we speculate that both the Chr14 and Chr17 are the potential to be related to SD in the mandarin fish. These results suggest the involvement of multiple chromosomes in sex relation, and provide supportive evidence to the polygenic SD in fishes (Zhang S. et al., 2019). In the coming future, the development of unisex male populations will be necessary for rapid improvement of the quality and quantity of the mandarin fish.

## Conclusion

In our present study, we generated a chromosomal-level genome assembly for the mandarin fish, which has been an economically important fish in China. Our genome assembly is high in quality, completeness, and accuracy based on multiple evaluations. Gene prediction, functional annotation, and evolutionary analysis provided novel insights into the genomic structure and mechanisms underlying food intake, SD, and prediction of new toxins. Our genome sequences will also offer a valuable genetic resource to support extensive fisheries and artificial breeding programs, and thereby allows for effective disease management, growth improvement, and discovering new drugs in the mandarin fish.

## Data Availability Statement

This Whole Genome Shotgun project of mandarin fish has been deposited in CNGBdb with accession number CNA0013732. Raw reads from Illumina sequencing are deposited in the CNGBdb with accession number CNS0204384. The genome assembly of mandarin fish has been deposited in the CNGB Nucleotide Sequence Archive (https://db.cngb.org/cnsa/) under the Project ID CNP0000961.

## Ethics Statement

The animal study was reviewed and approved by the Institutional Review Board on Bioethics and Biosafety of BGI, China (No. FT 18134). Written informed consent was obtained from the owners for the participation of their animals in this study.

## Author Contributions

XB, XY, and WD conceived and designed the research. WD and XZ performed the genome sequencing. XZ, JL, and YH performed data analyses and wrote the manuscript. WJ, ZC, and MW performed sample preparation. WD, XZ, XB, QS, and XY revised the manuscript. All authors approved submission of the manuscript for publication.

## Conflict of Interest

The authors declare that the research was conducted in the absence of any commercial or financial relationships that could be construed as a potential conflict of interest.

## Footnotes

1 http://www.cbs.dtu.dk/services/TMHMM/
