# Supplementary material for: A Chromosome-Level Genome Assembly of the Mandarin Fish (Siniperca chuatsi)
Source: Front Genet. 2021 Jun 23;12:671650. doi: 10.3389/fgene.2021.671650 (PMC8262678; doi:10.3389/fgene.2021.671650)
Supplement: Supplementary file 1 [file Presentation_1.zip › Suppl Tables & Figures.DOCX]

A chromosome-level genome assembly of the Mandarin fish (*Siniperca chuatsi*) provides into for its feeding habit of live preys

**Supplementary Materials**

**Table S1: Olfactory receptor** **reference sequences**

**Table S2: Accession numbers of the used 7,093 reference sequences for toxin prediction.**

**Table S3**: **Summary of sequenced reads**

| **Insert Size** | **Reads Length** | **Total Data(G)** |
| --- | --- | --- |
| 270bp | 150 | 138.35 |
| 500bp | 125 | 30.37 |
| 800bp | 125 | 23.62 |
| 2kb | 125 | 33.36 |
| 5kb | 125 | 36.98 |
| 10kb  20kb | 125  125 | 30.85  33.47 |
| **Total** |  | **327** |

**Table S4: 17-mer depth distribution**

| **kmer** | **kmer num** | **Kmer depth** | **genome size** | **used base** | **used read** | **X** |
| --- | --- | --- | --- | --- | --- | --- |
| 17 | 43888350480 | 59 | 743870347 | 50640404400 | 422003370 | 68.0769 |

**Table S5**. **Categorization of repetitive sequences.**

| Type | Repbase TEs | | TE protiens | | De novo | | Combined TEs | |
| --- | --- | --- | --- | --- | --- | --- | --- | --- |
|  | Length (Bp) | % in genome | Length (Bp) | % in genome | Length (Bp) | % in genome | Length (Bp) | % in genome |
| DNA | 25,142,867 | 3.31 | 3,835,322 | 0.50 | 59,425,392 | 7.83 | 73,325,280 | 9.66 |
| LINE | 29,263,268 | 3.85 | 25,214,038 | 3.32 | 94,464,231 | 12.44 | 105,937,778 | 13.96 |
| SINE | 1,522,203 | 0.20 | 0 | 0 | 1,084,634 | 0.14 | 2,517,642 | 0.33 |
| LTR | 7,682,854 | 1.01 | 3,663,965 | 0.48 | 33,625,638 | 4.43 | 38,275,146 | 5.04 |
| Other | 7,331 | 0.0009 | 0 | 0 | 0 | 0 | 7,331 | 0.0009 |
| Unknown | 0 | 0 | 0 | 0 | 19,146,692 | 2.52 | 19,146,692 | 2.52 |
| Total | 59,103,751 | 7.78 | 32,694,731 | 4.30 | 186,500,313 | 24.57 | 199,596,048 | 26.30 |

**Table S6: Summary statistics of gene annotation**

| **Method** | **gene set** | **number** | **average transcript length（bp)** | **average CDS length（bp)** | **average Exons per Gene** | **average exon length（bp)** | **average Intron length（bp)** |
| --- | --- | --- | --- | --- | --- | --- | --- |
| ***De novo*** | Augustus | 22331 | 15667.304 | 1608.441 | 9.32927 | 172.408 | 1687.8859 |
|  | Genscan | 27713 | 18933.796 | 1630.498 | 9.23747 | 176.509 | 2100.5598 |
| **Homolog** | *Danio_rerio* | 20042 | 12298.840 | 1609.944 | 8.61022 | 186.981 | 1404.5452 |
|  | *Oryzias_latipes* | 25435 | 9360.0232 | 1304.620 | 7.08425 | 184.158 | 1323.9756 |
|  | *Oreochromis_niloticus* | 25902 | 10818.721 | 1428.920 | 7.89541 | 180.981 | 1361.7459 |
|  | *Takifugu_rubripes* | 21704 | 11888.248 | 1515.439 | 8.46466 | 179.031 | 1389.5887 |
|  | *Gasterosteus_aculeatus* | 26693 | 9699.9238 | 1271.873 | 7.21811 | 176.206 | 1355.4040 |
| **Transcriptome** | Cufflinks | 20028 | 9656.5434 | 2405.208 | 9.14610 | 262.976 | 890.16091 |
| **GLEAN** | Total | 19904 | 19308.463 | 1878.251 | 10.5369 | 178.255 | 1827.6645 |

**Table S7:** **Summary statistics of gene functional annotation**

|  | **Number** | **Percentage (%)** |
| --- | --- | --- |
| InterPro | 17,545 | 88.14 |
| GO | 14,071 | 70.69 |
| KEGG | 16,679 | 83.79 |
| Swissprot | 18,220 | 91.53 |
| TrEMBL | 19,036 | 95.63 |
| Annotated | 19,059 | 95.75 |
| Unanotated | 845 | 4.24 |
| Total | 19,904 | 100 |

**Table S8: KEGG annotation of specific genes in mandarin fish**


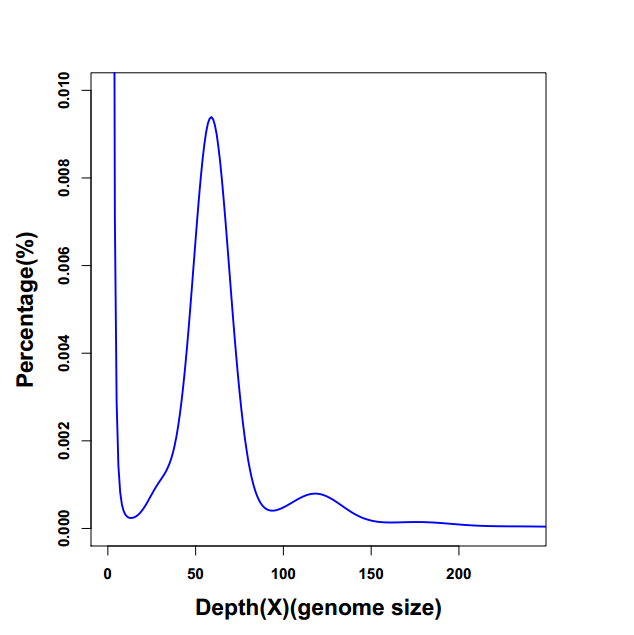


**Figure S1: K-mer (K-17) distribution in the mandarin fish genome**


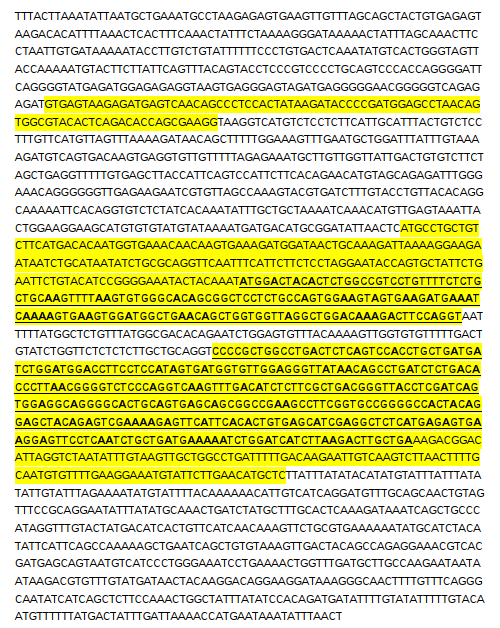


**Figure S2**: **Full length of leptin gene of mandarin fish**. Yellow areas indicate exons; The underlines represent coding sequences.


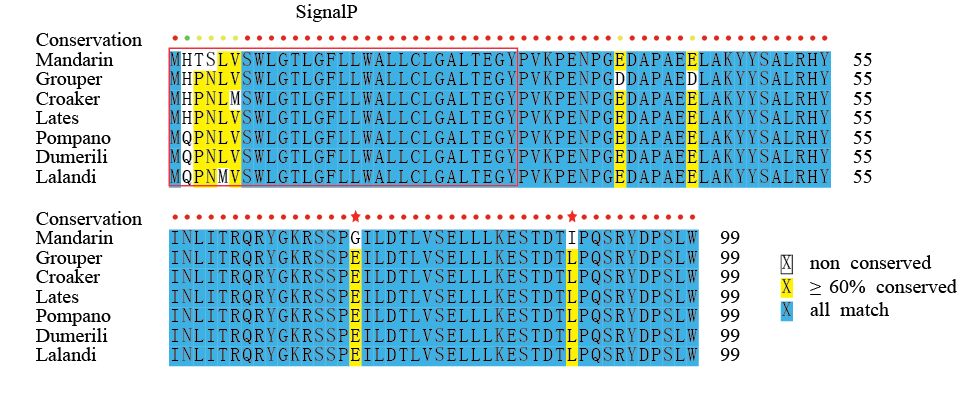


**Figure S3**: **ClustalX alignment of amino acid sequences of *npy* from seven species**. The red underlined represents signal peptides. The red asterisks indicated the mandarin fish specific mutation sites. Blue areas indicate conserved residues. Croaker: *Larimichthys crocea*, Dumerili: *Seriola dumerili*, Grouper: *Epinephelus coioides*, Lalandi: *Seriola lalandi dorsalis*, Lates: *Lates calcarifer*, Pompano: *Trachinotus ovatus*.


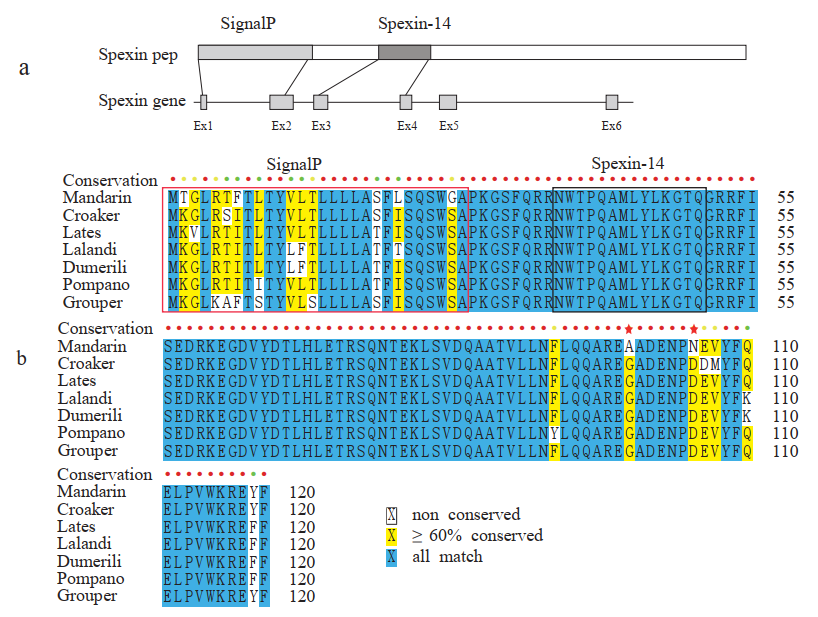


**Figure S4 (a) Intron–exon organization of mandarin fish *spexin* deduced from the full-length gene sequence.** Signal peptide is shaded in gray, and the mature peptide is shaded in black. Ex: exon. **(b) ClustalX alignment of amino acid sequences of *spexin* from seven species.** The red underlined represents signal peptides. The red asterisks indicated the mandarin fish specific mutation sites. Blue areas indicate conserved residues. Croaker: *Larimichthys crocea*, Dumerili: *Seriola dumerili*, Grouper: *Epinephelus coioides*, Lalandi: *Seriola lalandi dorsalis*, Lates: *Lates calcarifer*, Pompano: Trachinotus ovatus.


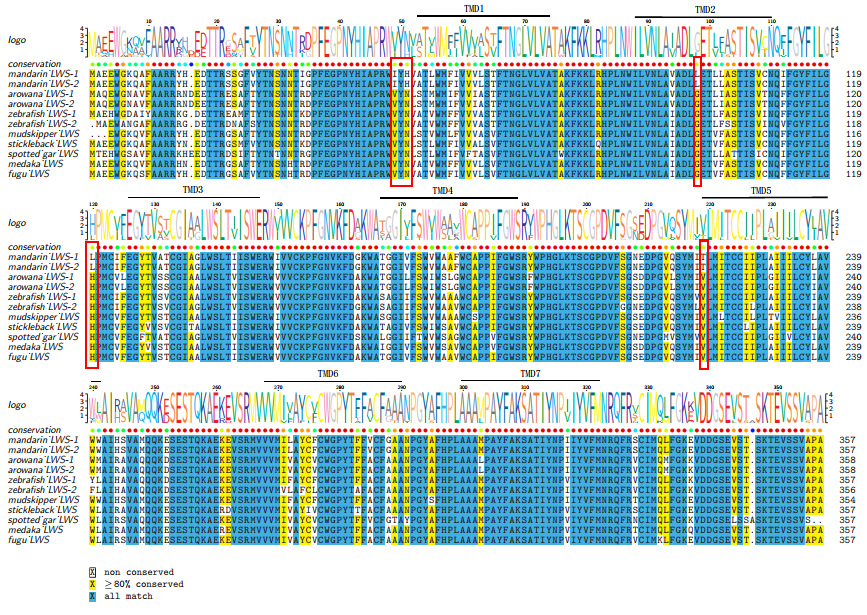


**Figure S5:** **LWS amino acid sequences from mandarin fish and seven other teleost fish.** The analysis was conducted in Mega and colorized by TEXshade. Amino acid changes are marked within the red boxes. The seven transmembrane domains, identified with TMHMM Server, are marked above the alignments as TMD1–7.


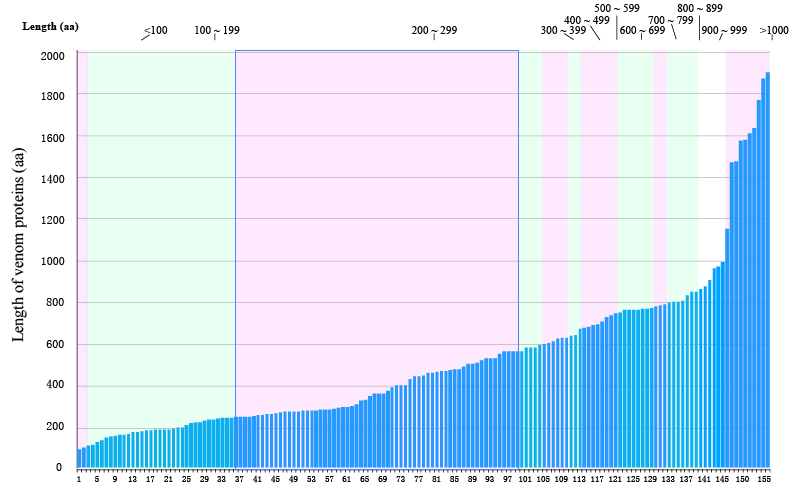


**Figure S6**. **Length distribution of the predicted venom genes from the mandarin fish genome.**


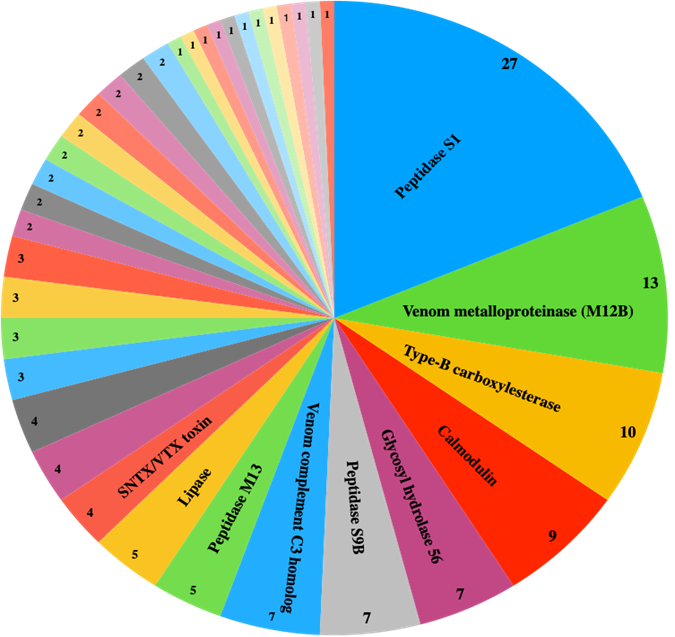


**Figure S7**. **Family distribution of the predicted venom genes from the mandarin fish genome.**
