# Supplementary figures and images for: A Chromosome-Level Genome Assembly of the Mandarin Fish (Siniperca chuatsi)
Source: Front Genet. 2021 Jun 23;12:671650. doi: 10.3389/fgene.2021.671650 (PMC8262678; doi:10.3389/fgene.2021.671650)

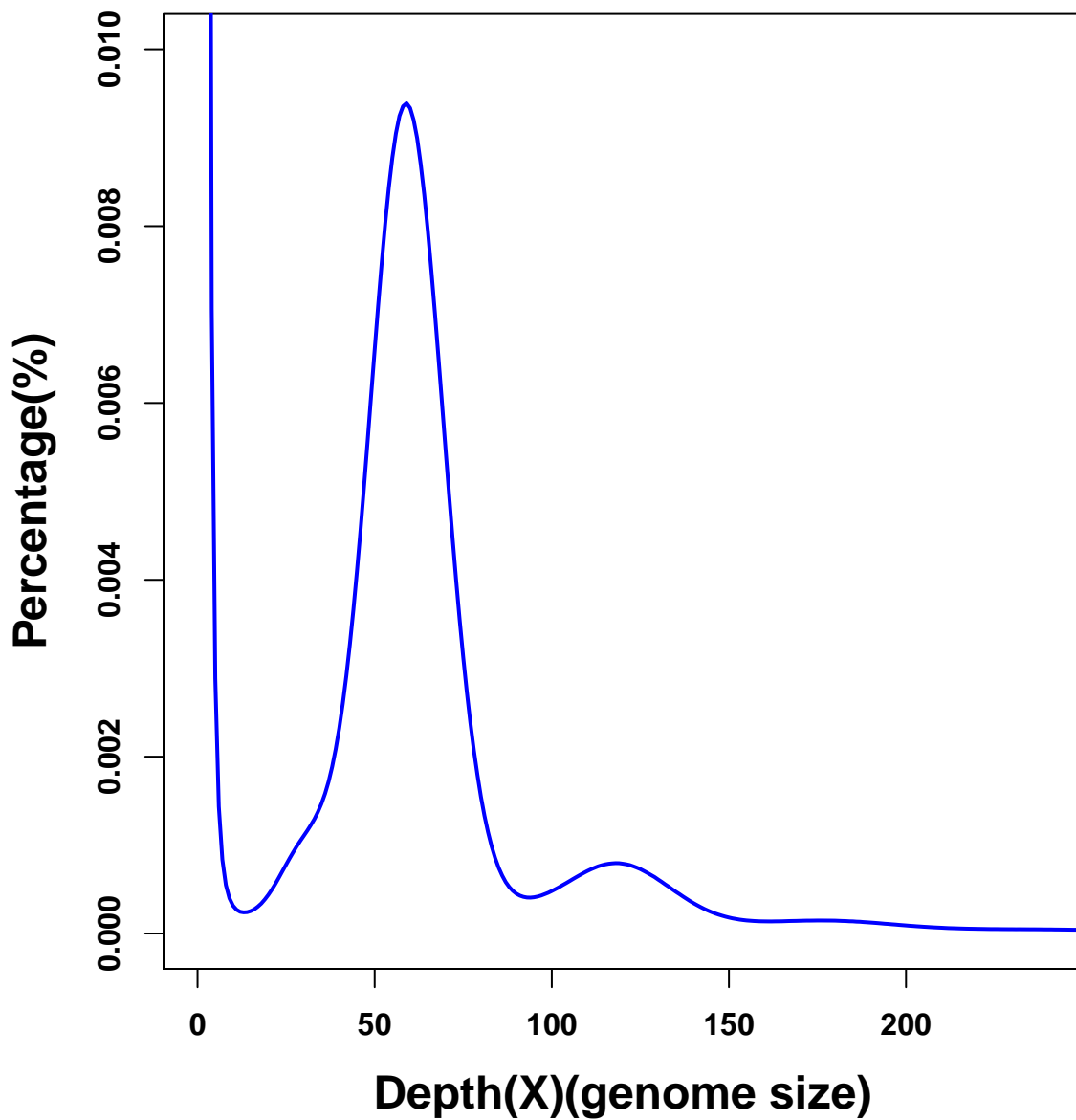

Supplement: Supplementary file 2 [file Data_Sheet_1.PDF]

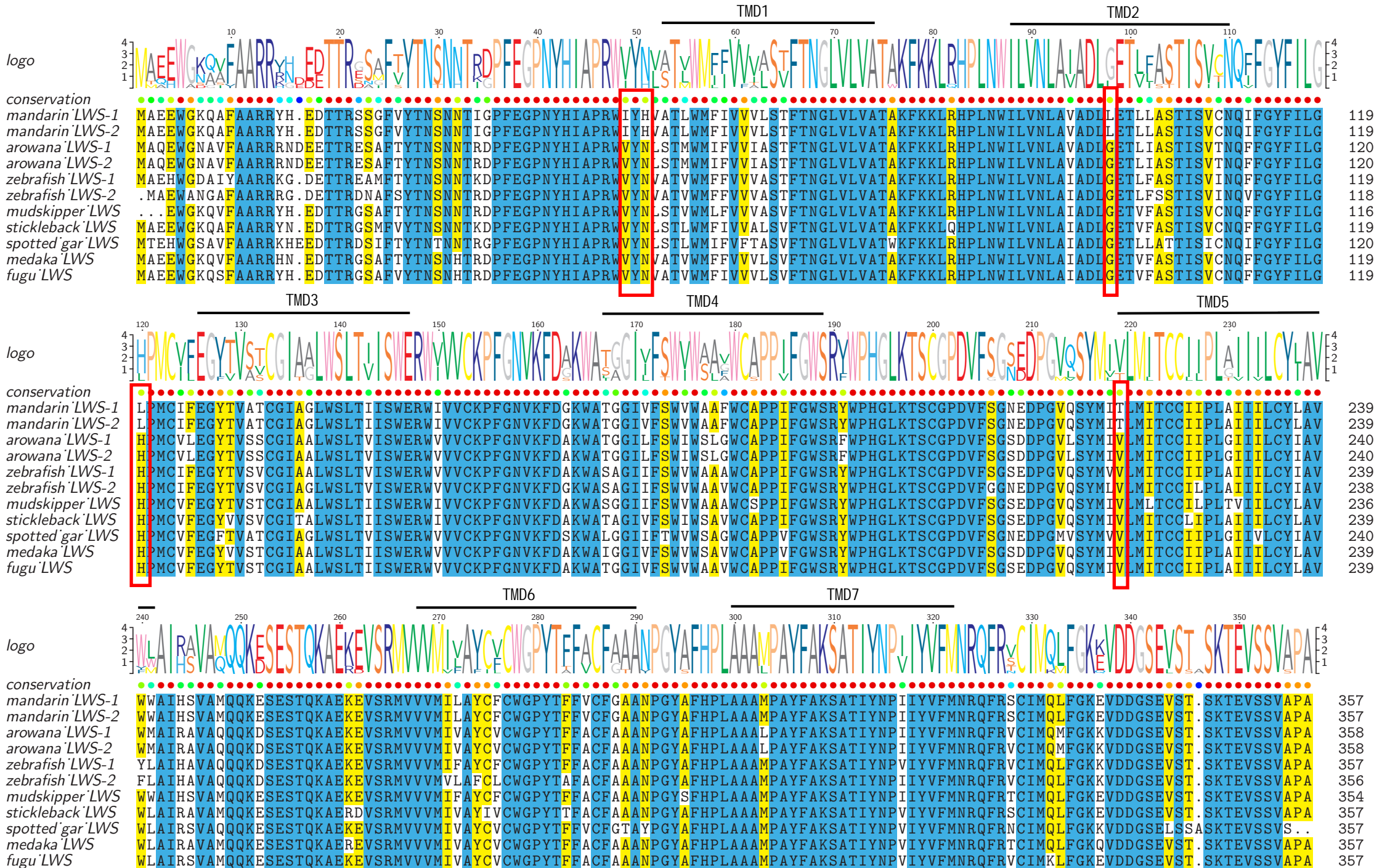

Supplement: Supplementary file 6 [file Data_Sheet_5.PDF]

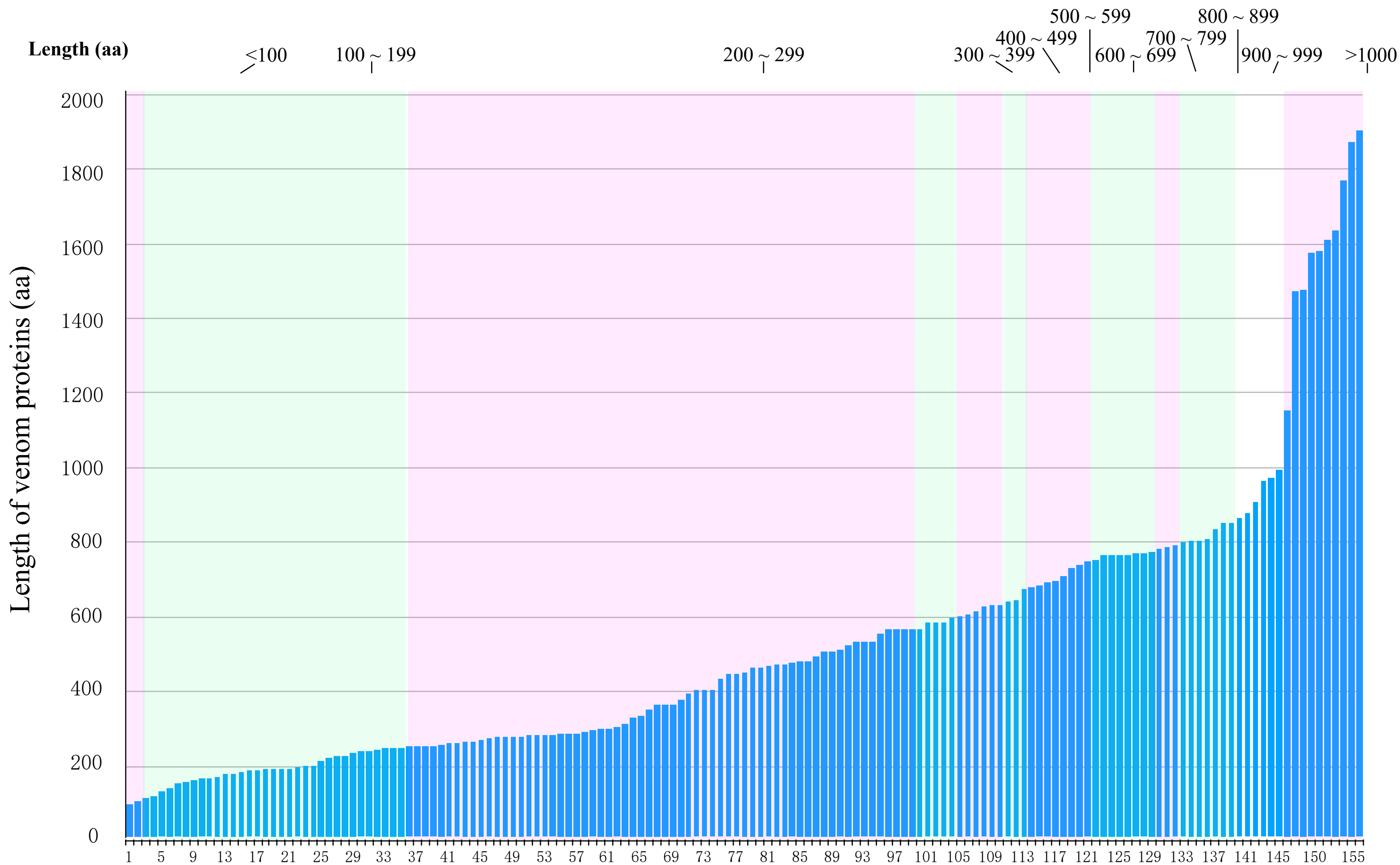

Supplement: Supplementary file 7 [file Data_Sheet_6.PDF]
