# Supplementary material for: A Chromosome-Level Genome Assembly of the Mandarin Fish (Siniperca chuatsi)
Source: Front Genet. 2021 Jun 23;12:671650. doi: 10.3389/fgene.2021.671650 (PMC8262678; doi:10.3389/fgene.2021.671650)
Supplement: Supplementary file 3 [file Data_Sheet_2.PDF]

TTTACTTAAATATTAATGCTGAAATGCCTAAGAGAGTGAAGTTGTTTAGCAGCTACTGTGAGAGT
AAGACACATTTTAAACTCACTTTCAAACTATTTCTAAAAGGGATAAAAACTATTTAGCAAACTTC
CTAATTGTGATAAAAATACCTTGTCTGTATTTTTTCCCTGTGACTCAAATATGTCACTGGGTAGTT
ACCAAAAATGTACTTCTTATTCAGTTTACAGTACCTCCCGTCCCCTGCAGTCCCACCAGGGGATT
CAGGGGTATGAGATGGAGAGAGGTAAGTGAGGGAGTAGATGAGGGGGAACGGGGGTCAGAG
AGATGTGAGTAAGAGATGAGTCAACAGCCCTCCACTATAAGATACCCCGATGGAGCCTAACAG
TGGCGTACACTCAGACACCAGCGAAGGTAAGGTCATGTCTCCTCTTCATTGCATTTACTGTCTCC
TTTGTTCATGTTAGTTTAAAAGATAACAGCTTTTTGGAAAGTTTGAATGCTGGATTTATTTGTAAA
AGATGTCAGTGACAAGTGAGGTGTTGTTTTTAGAGAAATGCTTGTTGGTTATTGACTGTGTCTTCT
AGCTGAGGTTTTTGTGAGCTTACCATTCAGTCCATTCTTCACAGAACATGTAGCAGAGATTTGGG
AAACAGGGGGGTTGAGAAGAATCGTGTTAGCCAAAGTACGTGATCTTTGTACCTGTTACACAGG
CAAAAATTCACAGGTGTCTCTATCACAAATATTTGCTGCTAAAATCAAACATGTTGAGTAAATTA
CTGGAAGGAAGCATGTGTGTATGTATAAAATGATGACATGCGGATATTAACTCATGCCTGCTGT
CTTCATGACACAATGGTGAAACAACAAGTGAAAGATGGATAACTGCAAAGATTAAAAGGAAGA
ATAATCTGCATAATATCTGCGCAGGTTCAATTTCATTCTTCTCCTAGGAATACCAGTGCTATTCTG
AATTCTGTACATCCGGGGAAATACTACAAAT**ATGGACTACACTCTGGCCGTCCTGTTTTCTCTG**
CTGCAAGTTTTAAGTGTGGGCACAGCGGCTCCTCTGCCAGTGGAAGTAGTGAAGATGAAAT
CAAAAGTGAAGTGGATGGCTGAACAGCTGGTGGTTAGGCTGGACAAAGACTTCCAGGTAAT
TTTTATGGCTCTGTTTATGGCGACACAGAATCTGGAGTGTTTACAAAAGTTGGTGTGTTTTTGACT
GTATCTGGTTCTCTCTCTTGCTGCAGGT**CCCCGCTGGCCTGACTCTCAGTCCACCTGCTGATGA**
TCTGGATGGACCTTCCTCCATAGTGATGGTGTTGGAGGGTTATAACAGCCTGATCTCTGACA
CCCTTAACGGGGTCTCCCAGGTCAAGTTTGACATCTCTTCGCTGACGGGTTACCTCGATCAG
TGGAGGCAGGGGCACTGCAGTGAGCAGCGGCCGAAGCCTTCGGTGCCGGGGCCACTACAG
GAGCTACAGAGTCGAAAAGAGTTCATTCACACTGTGAGCATCGAGGCTCTCATGAGAGTGA
AGGAGTTCCTCAATCTGCTGATGAAAAATCTGGATCATCTTAAGACTTGCTGAAAGACGGAC
ATTAGGTCTAATATTTGTAAGTTGCTGGCCTGATTTTTGACAAGAATTGTCAAGTCTTAACTTTTG
CAATGTGTTTTGAAGGAAATGTATTCTTGAACATGCTCTTATTTATATACATATGTATTTATTTATA
TATTGTATTTAGAAAATATGTATTTTACAAAAAACATTGTCATCAGGATGTTTGCAGCAACTGTAG
TTTCCGCAGGAATATTTATATGCAAACTGATCTATGCTTTGCACTCAAAGATAAATCAGCTGCCC
ATAGGTTTGTACTATGACATCACTGTTCATCAACAAAGTTCTGCGTGAAAAAATATGCATCTACA
TATTCATTCAGCCAAAAAGCTGAATCAGCTGTGTAAAGTTGACTACAGCCAGAGGAAACGTCAC
GATGAGCAGTAATGTCATCCCTGGGAAATCCTGAAAACTGGTTTGATGCTTGCCAAGAATAATA
ATAAGACGTGTTTGTATGATAACTACAAGGACAGGAAGGATAAAGGGCAACTTTTGTTTCAGGG
CAATATCATCAGCTCTTCCAAACTGGCTATTTATATCCACAGATGATATTTTGTATATTTTTGTACA
ATGTTTTTTATGACTATTTGATTAAAACCATGAATAAATATTTAACT
