# Supplementary material for: A Chromosome-Level Genome Assembly of the Mandarin Fish (Siniperca chuatsi)
Source: Front Genet. 2021 Jun 23;12:671650. doi: 10.3389/fgene.2021.671650 (PMC8262678; doi:10.3389/fgene.2021.671650)
Supplement: Supplementary file 4 [file Data_Sheet_3.PDF]

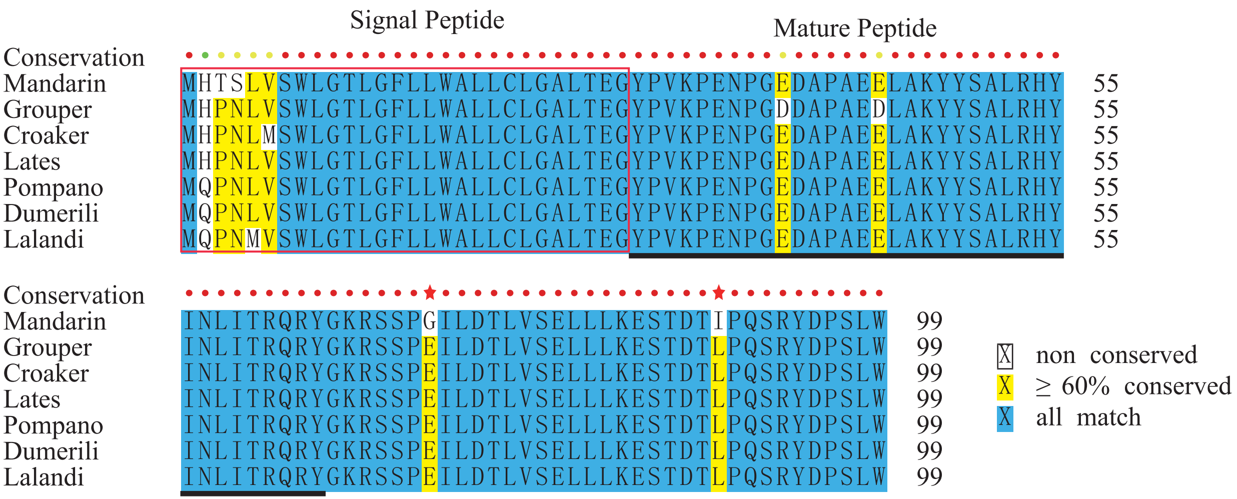
Signal Peptide
Mature Peptide
Conservation
Mandarin MHTSLVSWLGTLGFLLWALLCLGALTEGYPVKPENPGEDAPAEELAKYYSALRHY 55
Grouper MHPNLVSWLGTLGFLLWALLCLGALTEGYPVKPENPGDDAPAEDLAKYYSALRHY 55
Croaker MHPNLMSWLGTLGFLLWALLCLGALTEGYPVKPENPGEDAPAEELAKYYSALRHY 55
Lates MHPNLVSWLGTLGFLLWALLCLGALTEGYPVKPENPGEDAPAEELAKYYSALRHY 55
Pompano MQPNLVSWLGTLGFLLWALLCLGALTEGYPVKPENPGEDAPAEELAKYYSALRHY 55
Dumerili MQPNLVSWLGTLGFLLWALLCLGALTEGYPVKPENPGEDAPAEELAKYYSALRHY 55
Lalandi MQPNMVSWLGTLGFLLWALLCLGALTEGYPVKPENPGEDAPAEELAKYYSALRHY 55
Conservation
Mandarin INLITRQRYGKRSSPGILDTLVSELLLKESTDTIPQSRYDPSLW 99
Grouper INLITRQRYGKRSSPEILDTLVSELLLKESTDTLPQSRYDPSLW 99
Croaker INLITRQRYGKRSSPEILDTLVSELLLKESTDTLPQSRYDPSLW 99
Lates INLITRQRYGKRSSPEILDTLVSELLLKESTDTLPQSRYDPSLW 99
Pompano INLITRQRYGKRSSPEILDTLVSELLLKESTDTLPQSRYDPSLW 99
Dumerili INLITRQRYGKRSSPEILDTLVSELLLKESTDTLPQSRYDPSLW 99
Lalandi INLITRQRYGKRSSPEILDTLVSELLLKESTDTLPQSRYDPSLW 99
X non conserved
X ≥ 60% conserved
X all match
