# Supplementary material for: A Chromosome-Level Genome Assembly of the Mandarin Fish (Siniperca chuatsi)
Source: Front Genet. 2021 Jun 23;12:671650. doi: 10.3389/fgene.2021.671650 (PMC8262678; doi:10.3389/fgene.2021.671650)
Supplement: Supplementary file 5 [file Data_Sheet_4.PDF]

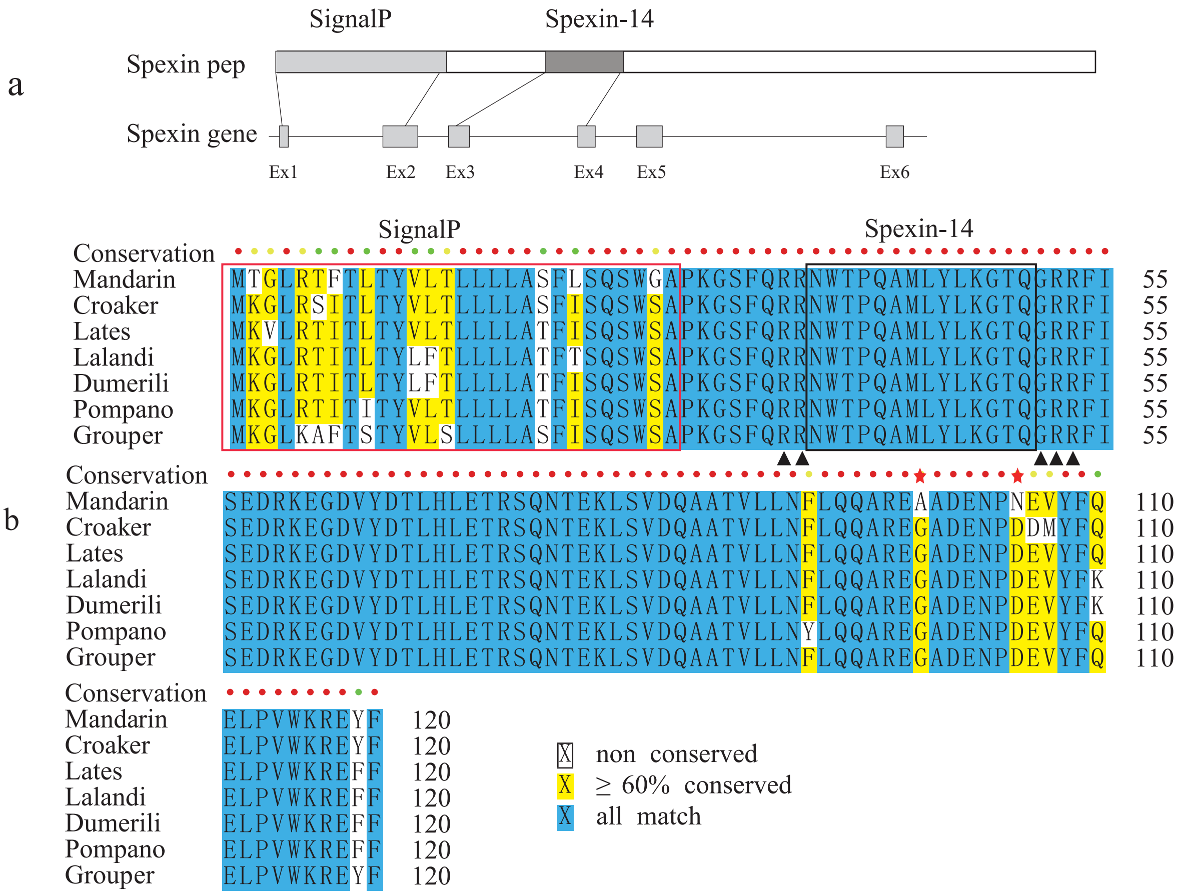
a
SignalP
Spexin-14
Spexin pep
Spexin gene
Ex1
Ex2
Ex3
Ex4
Ex5
Ex6
b
SignalP
Spexin-14
Conservation
Mandarin MTGLRTFTLTYVLTLLLLASFLSQSWGAPKGSFQRRNWTPQAMLYLKGTQGRRFI 55
Croaker MKGLRSITLTYVLTLLLLASFISQSWSAPKGSFQRRNWTPQAMLYLKGTQGRRFI 55
Lates MKVLRTITLTYVLTLLLLATFISQSWSAPKGSFQRRNWTPQAMLYLKGTQGRRFI 55
Lalandi MKGLRTITLTYLFTLLLLATFTSQSWSAPKGSFQRRNWTPQAMLYLKGTQGRRFI 55
Dumerili MKGLRTITLTYLFTLLLLATFISQSWSAPKGSFQRRNWTPQAMLYLKGTQGRRFI 55
Pompano MKGLRTITITYVLTLLLLATFISQSWSAPKGSFQRRNWTPQAMLYLKGTQGRRFI 55
Grouper MKGLKAFTSTYVLSLLLLASFISQSWSAPKGSFQRRNWTPQAMLYLKGTQGRRFI 55
Conservation
Mandarin SEDRKEGDVYDTLHLETRSQNTEKLSVDQAATVLLNFLQQAREAADENPNEVYFQ 110
Croaker SEDRKEGDVYDTLHLETRSQNTEKLSVDQAATVLLNFLQQAREGADENPDDMYFQ 110
Lates SEDRKEGDVYDTLHLETRSQNTEKLSVDQAATVLLNFLQQAREGADENPDEVYFQ 110
Lalandi SEDRKEGDVYDTLHLETRSQNTEKLSVDQAATVLLNFLQQAREGADENPDEVYFK 110
Dumerili SEDRKEGDVYDTLHLETRSQNTEKLSVDQAATVLLNFLQQAREGADENPDEVYFK 110
Pompano SEDRKEGDVYDTLHLETRSQNTEKLSVDQAATVLLNYLQQAREGADENPDEVYFQ 110
Grouper SEDRKEGDVYDTLHLETRSQNTEKLSVDQAATVLLNFLQQAREGADENPDEVYFQ 110
Conservation
Mandarin ELPVWKREYF 120
Croaker ELPVWKREYF 120
Lates ELPVWKREFF 120
Lalandi ELPVWKREFF 120
Dumerili ELPVWKREFF 120
Pompano ELPVWKREFF 120
Grouper ELPVWKREYF 120
X non conserved
X ≥ 60% conserved
X all match
