# Supplementary material for: A Chromosome-Level Genome Assembly of the Mandarin Fish (Siniperca chuatsi)
Source: Front Genet. 2021 Jun 23;12:671650. doi: 10.3389/fgene.2021.671650 (PMC8262678; doi:10.3389/fgene.2021.671650)
Supplement: Supplementary file 9 [file Data_Sheet_8.PDF]

超星·期刊

《淡水渔业》1988年第5期

鳜幼鱼摄食行为的初步分析*

吴 遵 霖

（湖北省水产研究所）

W·Hardy

（美国西雅图华盛顿大学）**

提要 把鳜幼鱼的摄食行为分解成反应、注视、跟进、袭击、咬住和吞噬六个连续的系列动作，并试用"嗜食度"来直观定量地分析鳜幼鱼对真鱼、假鱼与非鱼三类十五种试验饵料的嗜好程度。初步认为鳜幼鱼主要依靠视觉发现和辨别具有鱼形的活鱼或新鲜死鱼，然后摄取。

鳜（*Sjniperca chuatsi*）是原产中国的鮨科（Serranidae）鱼类，具有极高的食用经济价值。但是这种鱼从仔鱼"开食"起，终身以鲜活鱼虾为食，食性特殊，在很大程度上限制了其种群的繁衍和大规模养殖。1986年湖北省科委对外交流中心邀请美国西雅图华盛顿大学与我方组成"鳜鱼饵料研究"课题组，进行合作研究，试图以配合饵料代替活鱼虾。现把初步进行的摄食行为分析报告如下。

材料与方法

鳜幼鱼是经人工催产、孵化后，在室内养成。供试幼鱼全长50～80毫米，1～2月龄。每次供试数量7～10尾。

试验饵料有四种配方，主要成分是鱼糜及鱼肉浆加大麦玉米淀粉，占50～60%，其次有鱼油、纤维素、维生素和矿物质等。饵料中分别加有四种引诱物质，代号C、TV、K和甘氨酸、丙氨酸，前三种为美方提供，甘氨酸丙氨酸为日本学者称为的鱼类"摄饵促进物质"。原料粒度60～100目，加水40%，充分拌匀，用手捏成面团状，冷藏待用。

活鱼饵料为食蚊鱼（*Gambasia affini*全长15～50毫米，活鱼卵（为此种鱼的怀卵巢），以及此种鱼的鱼头或鱼尾。塑料鱼为模拟该鱼体外形，用无色透明塑料制成。死鱼为将该鱼致死的鱼。蚯蚓为日本赤子爱胜蚓"大平二号"幼蚓。蜗牛肉为"福寿螺"肉足。

试验方法为直接观察，有三种方法。

方法一：用24×44×17厘米玻璃水箱，盛20升水，底层铺细沙并植入水生植物。用气泵充气，保持水中溶氧6～7毫克/升。放入试验鳜，适应性饲养一周后供试。各种试验食物或模拟物用3～5根（相当细度3～5支）透明尼龙丝系牢，活鱼即系在下颌上。活鱼模拟物长度约为鳜鱼全长的70%。用手提着细尼龙丝，放在离鳜鱼20～25厘米处，朝鳜鱼抖动并模仿活鱼游弋，移动速度约5～10厘米/秒。另外还放在箱外，紧贴水箱壁，运动状态同箱内。

拍摄和记录试验食物放入后，鳜鱼的运动反应。根据观察，鳜鱼摄食行为是一"行为系列"。试把这一系列运动反应分解成六个动作等级（0级—5级）。即：(0)无反应。试验食物放入后，鳜鱼仍在隐蔽

*本文承倪达书研究员、杨永铨副局长审阅。参加试验的还有张鑫昌、张建枝等，谨致谢忱。

**W·Hardy博士是美国西雅图华盛顿大学教授、鱼类营养学家。

超星·期刊

处，眼球不动，视而不见，头不朝向食物。（1）注视反应。鳜鱼双眼一起转动，头朝向食物呈注视状，但尚未游动。（2）跟进反应。鳜鱼明显朝食物方向慢慢游动，接近食物，跟食而进，游动距离超过鱼与食距离的1/3以上。（3）袭击反应。游至离食物5～8厘米处，鳜鱼往往作极短暂的停留，头部偏左或右，背鳍竖起，尾鳍钩向一侧并对食物再次辨别后，胸腹鳍摆动加快，瞬间突然向食物袭击。（4）咬住反应。袭击后，鳜鱼把食物全部咬住或咬下一部分，无论咬后吐出或吞下均为咬住反应。（5）吞噬反应。鳜鱼吞进食物2分钟内没有吐出，被认定已经吞下。

这六个动作按顺序进行，全部完成即完成摄食行为。但很可能只完成到某一动作而中止。很明显越能完成后面的动作，鱼对该食物嗜好性越高。从放入食物到发生运动反应或不反应延时到2分钟称为试验一次。各种食物进行的试验次数一般为5次，考虑有多于或少于5次的，还必需除以总试验次数。鱼对某食物的运动反应以最后的动作为准，所需时间为反应时间，显然鱼对某食物嗜好性越强，反应时间越短，每一动作的间隔时间越短。

试验鱼在试验前2～3天不喂食，鱼对食物的嗜好性与饥饿程度呈正相关。同时在鳜鱼的适温范围内（26～30℃），与水温也呈正相关。

为了定量比较某鱼类对各种食物嗜好性，作者试用“鱼嗜食度”表示某鱼类对各种食物的嗜好，并激起摄食运动反应的程度。它仅适用于不同鱼类和不同食物间，在同样试验条件下的相互比较。其表达式如下：

$$鱼嗜食度（D）=\Sigma\frac{N\cdot C\cdot Wt}{iN\cdot F\cdot At}K$$

式中N—试验次数．iN—试验总次数。Wt—水温。C—级数（或加权数），六种运动反应动作依次为0、1、2、3、4、5。

F—饱食率即饥饿程度。对于鳜鱼的观察表明，饱食的幼鱼5天不进食的观察表明，饱食的幼鱼5天不进食即不可逆地饿死，因此这里把饿三天的饱食率定为0.4．饿两天为0.6，饿一天为0.8。

At—平均反应时间。即每次试验平均持续时间。

K—品种系数，假定鳜鱼品种系数是0.01。其他品种依鳜鱼相同方法折算。

方法二：用黄色氧化汞眼膏遮盖鳜幼鱼两侧或一侧眼睛，用不遮眼的鱼作为对照。同时放进上述水箱中，观察鳜鱼的摄食行为。另外再用白色医用凡士林把上述鳜幼鱼的两侧鼻囊孔塞起来，同样观察其摄食行为。

方法三：用两个2000毫升大烧杯，装满净水和两尾鳜幼鱼，在其中一烧杯中混入河底黑色污泥，使混水透明度在8厘米以下，适应一段时间后，同时投进活鱼各两尾，观察被捕食的情况。

结果及分析

一．试验食物对象分为三类：第一类是真鱼，即其质是鱼，有鱼的化学气味物质，但有的如鱼卵无鱼的形状。而箱外的真鱼隔一层玻璃，气味不能刺激鳜鱼，第二类为假鱼，其质不是鱼，但模拟鱼的形态。有的饵料鱼是真头尾，躯干是饵料做的，接近真鱼。第三类不是鱼，除蜻蜓幼虫，体长、摆动、游泳类似鱼形外，其他无鱼形。

按方法一共进行二次摄食行为分析试验。结果分别列于表1、2。第一次试验鳜幼鱼饥饿1天，摄食运动反应只分为五种（无吞进），为初试。第二次饥饿两天为正式试验。表1、2可见：（1）活鱼和死鱼嗜食度最高，并且活鱼＞死新鲜鱼＞死腐败鱼。其次是有真鱼头尾饵料鱼身的鱼。说明

超星·期刊

表1　鳜鱼摄食行为第一次分析试验结果*　（水温29.5°C）

行为分析 / 试验食物对象		行为动作系列反应次数 无反应（0）	注视（1）	跟进（2）	袭击（3）	咬后吐出（4）	平均反应时间（分）	鱼嗜食度
真鱼	死鱼（已腐败）		3	3	2	2	0.5～0.9	1.48
真鱼	真鱼在箱外（隔玻璃）		5				0.8	0.46
假鱼（具鱼形）	塑料鱼	1	4	4			0.6～0.8	0.75
假鱼（具鱼形）	干鱿鱼片		5	8			0.6～0.9	0.62
假鱼（具鱼形）	真鱼头尾饵料鱼身（外沾甘氨酸）		7	5	3	3	0.5～0.8	1.72
非鱼	蚯蚓	2						0

*饱食率0.8，嗜食度依前表达式计算（下同）

表2　鳜幼鱼摄食行为第二次分析试验结果　（水温28°C饱食率0.6）

行为分析 / 试验食物对象		行为动作系列反应次数 无反应（0）	注视（1）	跟进（2）	袭击（3）	咬住（4）	吞进（5）	平均反应时间（分）	鱼嗜食度
真鱼	活鱼						5	0.2	11.67
真鱼	死鱼（新鲜）					1	4	0.3	7.46
真鱼	鲜鱼卵块		3	2				0.8～0.9	0.78
真鱼	真鱼在箱外（隔玻璃）			3	2			0.9	1.20
假鱼（具鱼形）	塑料鱼		1	4				0.8	1.05
假鱼（具鱼形）	全饵料鱼 2号配方	2	4	3				0.9	0.61
假鱼（具鱼形）	全饵料鱼 6号配方		3	1	1			0.8	0.93
假鱼（具鱼形）	全饵料鱼 5号配方	3	2					0.9	0.21
假鱼（具鱼形）	全饵料鱼 4号配方＋丙氨酸	2	1	1				0.9	0.31
假鱼（具鱼形）	全饵料鱼 4号配方＋甘氨酸		3	2				0.8	0.82
假鱼（具鱼形）	真头尾饵料鱼身			4			1	0.3～0.6	2.80
非鱼	蚯蚓	3	2					0.9	0.20
非鱼	螺肉		3	2				0.8	0.82
非鱼	蜻蜓幼虫			4	1			0.6	1.72
非鱼	塑料圈	4	1					0.7	0.13

既有鱼的形态刺激，又有新鲜血气味刺激，引起鳜幼鱼摄食行为反应最强。（2）具有鱼形态的箱外鱼、塑料鱼、真头尾饵料鱼比不具有鱼的形态的（即使是真鱼鲜卵巢）嗜食度都高得多。同属非鱼类比较，类似鱼形的蜻蜓幼虫比其他物嗜食度也高得多，这说明感受鱼的形态刺激的视觉比感受鱼的气味物质刺激的嗅觉、味觉或其他感觉引起摄食反应要强。（3）表1、2的结果基本是一致的。但是表2与表1相比，各类试验食物的嗜食度要高些，其原因是第二次试验鱼饥饿比第一次试验多一天即饱食率小0.2.两次试验水温相差不大（15℃），对嗜食度影响也没有饱食度大。（4）试验食物含有不同的引诱物质的几类之间，嗜食度并无显著差别，可见在本试验中，这些所谓对摄饵有促进作用的引诱物质并未改变嗜食度。

二．基于对鳜鱼摄食行为主要依靠视觉

超星·期刊

《淡水渔业》1988年第5期

长吻鮠与大鳍鳠的含肉率及鱼肉营养成分的比较研究

陈定福　何学福　周启贵

（西南师范大学生物系）

提　要　本试验定量测定了长吻鮠、大鳍鳠的含肉率及鱼肉的主要营养成分，分析了肌肉水解液中氨基酸及肌肉游离氨基酸的含量，比较、评价了两种鱼的营养价值。结果表明，长吻鮠优于大鳍鳠。

长吻鮠（*Leiocassis Longiros Tris* GünTher）（地方名江团）和大鳍鳠（*Hemibagrus macropterus* Bleeker）属鲶形目鮠科鱼类。它们体无鳞、骨刺少、肉质细嫩、味鲜美，成为长江流域的重要经济鱼类。尤其是长吻鮠个体大，素为名贵鱼种、上等佳肴，而大鳍鳠具有种群数量大、经济价值较高的优点。1981年以来，西南师大生物系等单位将长吻鮠人工繁殖和移养驯化成功，并开展了试验性养殖。为了比较、评价它们的营养价值，我们于1987年3～6月对长吻鮠和大鳍鳠进行了含肉率的测定及鱼肉（肌肉）营养成分的生化分析，以便进行营养学的评价。

表1　研究材料

鱼别	体长（cm）	体重（g）	尾数	采集地	1987年采集月、日
长吻鮠	27.9—30	308—376	6(♀3,♂3)	我系养鱼池	3.6;4.5;6.5
	29—32	315—400	8(♀5,♂3)	嘉陵江北碚江段	3.6;4.5;6.5
大鳍鳠	30—39	190—380	6(♀3,♂3)	我系养鱼池	3.5;4.5;6.5
	29.2—36	182—354	6(♀3,♂3)	嘉陵江北碚江段	3.5;4.5;6.5

* 我系罗泉、张跃光、魏刚、王德寿等同志作了部分工作，在此致谢。

的初步认识，进行了方法二和三的试验。结果是当用黄色氧化汞眼膏遮盖一侧眼睛后，鳜鱼捕食活鱼的速度比对照稍慢2～3秒，饥饿一天，从发现活鱼到最后吞下约15～20秒。可以说游动、隐蔽、发现、辨别与捕食基本正常，影响不大。但遮盖两侧眼睛后，鳜鱼明显表现不安、呆痴笨拙，只能用头挨着水箱壁摸索游动，丧失捕食能力，把活鱼放下去半小时仍未被吃，但当抹去双眼遮盖，鳜鱼很快恢复活泼状态并迅速发现与捕食活鱼。为了防止遮盖不严，在以后的试验里，用浓硫酸烧坏双眼角膜，也看到与遮盖同样的现象，但经长时间（半月）适应后有了好转，但捕食能力仍比不上正常鱼。

当用白色凡士林把鳜鱼两侧鼻孔封严，无论游动还是发现，捕食和对照鱼没有显著差别。

在用方法三的试验中，用河泥把水弄混意欲模拟天然鳜鱼苗生活的水环境。投入两尾鳜鱼和两尾活鱼苗，结果一尾活鱼半小时才被吃掉，另一尾2小时还没有被食。作为对照的清水玻璃烧杯中，放入10分钟后检查，活鱼苗已被鳜鱼吃完。
