# Supplementary material for: A Chromosome-Level Genome Assembly of the Mandarin Fish (Siniperca chuatsi)
Source: Front Genet. 2021 Jun 23;12:671650. doi: 10.3389/fgene.2021.671650 (PMC8262678; doi:10.3389/fgene.2021.671650)
Supplement: Supplementary file 8 [file Data_Sheet_7.PDF]

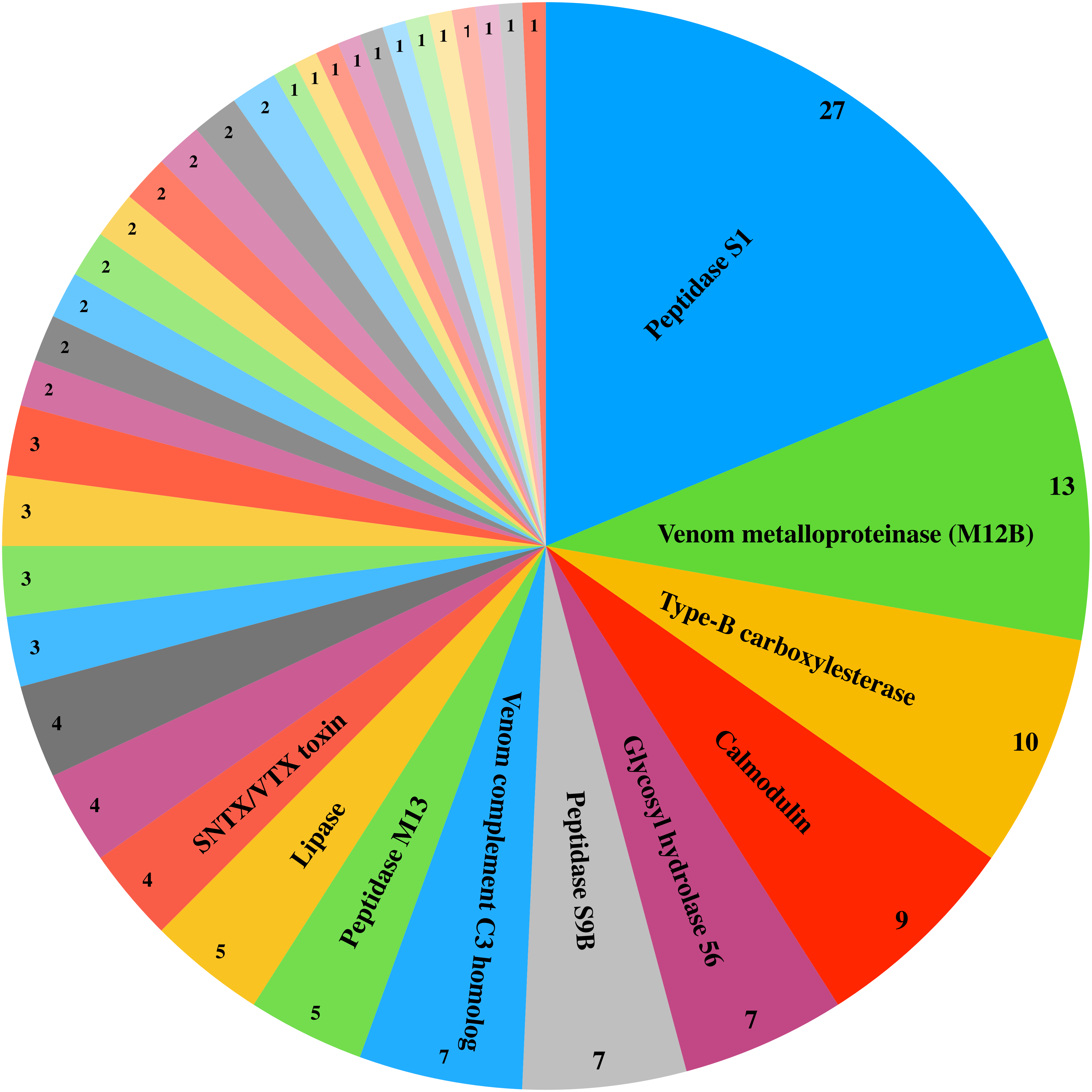

Peptidase S1
27
Venom metalloproteinase (M12B)
13
Type-B carboxylesterase
10
Calmodulin
9
Glycosyl hydrolase 56
7
Peptidase S9B
7
Venom complement C3 homolog
7
Peptidase M13
5
Lipase
5
SNTX/VTX toxin
4
4
4
3
3
3
3
2
2
2
2
2
2
2
2
2
2
1
1
1
1
1
1
1
1
1
1
1
1
